# Single-cell transcriptome sequencing allows genetic separation, characterization and identification of individuals in multi-person biological mixtures

**DOI:** 10.1038/s42003-023-04557-z

**Published:** 2023-02-20

**Authors:** Lucie Kulhankova, Diego Montiel González, Eric Bindels, Daniel Kling, Manfred Kayser, Eskeatnaf Mulugeta

**Affiliations:** 1 Department of Genetic Identification, Erasmus MC, University Medical Center Rotterdam, Rotterdam, the Netherlands; 2 Department of Hematology, Erasmus MC, University Medical Center Rotterdam, Rotterdam, the Netherlands; 3 Department of Forensic Genetics and Toxicology, National Board of Forensic Medicine, Linkoping, Sweden; 4 Department of Cell Biology, Erasmus MC, University Medical Center Rotterdam, Rotterdam, the Netherlands; 5 Present Address: Princes Maxima Center for Pediatric Oncology, Utrecht, the Netherlands

**Keywords:** Transcriptomics, Genetic variation

## Abstract

Identifying individuals from biological mixtures to which they contributed is highly relevant in crime scene investigation and various biomedical research fields, but despite previous attempts, remains nearly impossible. Here we investigated the potential of using single-cell transcriptome sequencing (scRNA-seq), coupled with a dedicated bioinformatics pipeline (De-goulash), to solve this long-standing problem. We developed a novel approach and tested it with scRNA-seq data that we *de-novo* generated from multi-person blood mixtures, and also *in-silico* mixtures we assembled from public single individual scRNA-seq datasets, involving different numbers, ratios, and bio-geographic ancestries of contributors. For all 2 up to 9-person balanced and imbalanced blood mixtures with ratios up to 1:60, we achieved a clear single-cell separation according to the contributing individuals. For all separated mixture contributors, sex and bio-geographic ancestry (maternal, paternal, and bi-parental) were correctly determined. All separated contributors were correctly individually identified with court-acceptable statistical certainty using *de-novo* generated whole exome sequencing reference data. In this proof-of-concept study, we demonstrate the feasibility of single-cell approaches to deconvolute biological mixtures and subsequently genetically characterise, and individually identify the separated mixture contributors. With further optimisation and implementation, this approach may eventually allow moving to challenging biological mixtures, including those found at crime scenes.

## Abstract (web summary)

A method is developed by combining scRNA-sequencing and a bioinformatics pipeline to separate individuals in multi-person biological mixtures, and, subsequently, determine the ancestry and sex of the separated individuals and genetically identify them.

## Introduction

Genetic characterization and individual genetic identification of persons who contributed to biological mixtures is relevant in different areas of science and society. Biological mixtures with a contribution of more than one individual are often collected at crime scenes. In cases with known perpetrators, individual genetic identification can pinpoint a perpetrator via comparative forensic DNA profiling^1^, while in those with unknown perpetrators, genetic characterization (such as on sex, biogeographic ancestry) can provide investigative leads to help finding the unknown perpetrator^1^. Successful genetic characterization and identification of individuals from mixed biomaterials starts with accurate mixture deconvolution, i.e., the separation of the mixed biomaterial according to the individual contributors, which is the most crucial, but at the same time most difficult step. Despite various attempts based on different methodologies, limitations in deconvoluting biological mixtures remain one of the major challenges of forensic DNA analysis^2–6^. Moreover, mixture separation is also relevant in other areas of biomedical research and applications, e.g., for detecting and resolving contamination in widely used cell, tissue and organoid cultures.

Currently, the most common technique used in forensic mixture deconvolution is differential lysis^7^, which is applied in mixtures involving semen cells of the male perpetrator and epithelial cells of the female victim typically encountered in sexual assault cases by analyzing vaginal swabs. However, differential lysis often results in incomplete separation of the male and female DNA fractions. In consequence, the resulting autosomal short tandem repeat (STR) profile still shows a mixture of alleles from the female victim and the male perpetrator. This makes it difficult, and often impossible, to single-out the STR-profile of the male perpetrator from the mixed DNA profile, even if the STR-profile of the female victim is known from reference DNA analysis^8^. Targeting the male-specific portion of the Y-chromosome offers help as it allows to specifically analyse male-specific STRs in the mixture and works in mixtures with large access of female DNA as in material from sexual assault cases^9^. However, forensic Y-STR profiling comes with the disadvantage that it most often cannot differentiate between paternally related men who typically share the same Y-STR profile. In consequence, the match probability obtained for the male suspect also applied for his paternal male relatives, and thus, conclusions cannot be drawn on the individual level as needed in court^9^. Methods for deconvoluting mixed autosomal STR-profiles obtained from mixed stains with the help of statistical methods, such as probabilistic genotyping, are available^10–13^, but their success is limited and depends on many factors^12,13^. Because of its quantitative nature, the use of next generation sequencing (NGS), also referred as massively parallel sequencing (MPS), for forensic STR-profiling provides some improvement in deconvoluting mixed STR-profiles, but its success is mainly restricted to less complex mixtures such as those of two persons^1^. Moreover, differential lysis is not suitable for resolving mixtures of semen from different men and not for mixtures involving no sperm cells at all.

Another major disadvantage of the current methods is that they aim to separate the mixed DNA profile rather than separating the mixed sample according to its contributors prior to DNA profiling. A potentially more promising mixture separation strategy would be to first separate the biological mixture according to the individual contributions, so that subsequent DNA analysis for genetic identification or characterization of the separated individual contributors becomes a technically less challenging single-source analysis. Recently, few methods for separating cells from a mixture prior to forensic STR-analysis have been tested for the purpose of forensic mixture deconvolution, such as DEPArray^tm^ ^14–16^, laser capture microdissection^17^, or FACS^18,19^. The main disadvantage of DEPArray^tm^ and laser capture microdissection is the low number of cells the techniques can separate. The lower the number of separated cells, the more likely it is to miss a minor contributor to the mixture. Although the number of cells can be increased by use of FACS, which requires fluorescent differences between separable cell types, FACS does not work for mixtures of the same cell type or cell types that cannot be fluorescently separated.

An overall drawback that unifies all currently available methods for mixture deconvolution is that, based on the limited amounts of DNA obtained from crime scenes, only partial STR-profiles are typically generated^14–16,18–20^. Because of the limited number of STRs included in commercial STR kits used in forensic practice, the match probabilities resulting from partial STR-profiles often are not high enough for concluding individual identification with the necessary statistical certainty accepted by the court. Increasing the number of STR markers in forensic STR kits is technically challenging; particularly for kits based on widely applied fluorescently labelled multiplex PCR and capillary electrophoresis (CE). Although targeted MPS can increase the number of STR markers relative to CE-analysis, in case such commercial kits become available in the future, sequencing STRs remains a challenge due to enzyme problems with sequencing repetitive DNA. Notably, this limitation is absent when it comes to single nucleotide polymorphisms (SNPs), which not only allow individual genetic identification but also genetic characterization of individuals^1,21,22^. Moreover, SNPs can easily be genotyped simultaneously in large numbers with targeted or non-targeted MPS technologies.

In recent years, several single-cell sequencing technologies involving large-scale genome, epigenome, and transcriptome sequencing have emerged and are revolutionizing biological and biomedical research and applications^23^. Single-cell sequencing techniques allow pre-labelling cells prior to large-scale sequencing and deliver ample amounts of SNP data for subsequent analysis. In principle, such single-cell sequencing technologies are expected to overcome the limitations of currently used methods for mixture deconvolution. However, to the best of our knowledge, high-throughput single-cell sequencing has not been applied as of yet for mixture deconvolution with subsequent genetic characterization and individual genetic identification of the separated contributors.

Here, we introduce a novel approach based on single-cell transcriptome sequencing with a dedicated bioinformatics pipeline that, by analysing multi-person biological mixtures, achieves genetic separation of the individual contributors as well as genetic characterization and individual genetic identification of the separated contributors, and additionally, determines the tissue of origin of biological mixtures. In this proof-of-principle study, we introduce our approach with and a dedicated bioinformatics pipeline and provide the first validation results using de novo generated scRNA-seq datasets from multi-person blood mixtures, and in silico generated mixtures from publicly available individual scRNA-seq datasets, involving different numbers of contributors with different bio-geographic ancestries and different ratios of the individual contributions.

## Results

### Bioinformatics pipeline

**Fig. 1:**
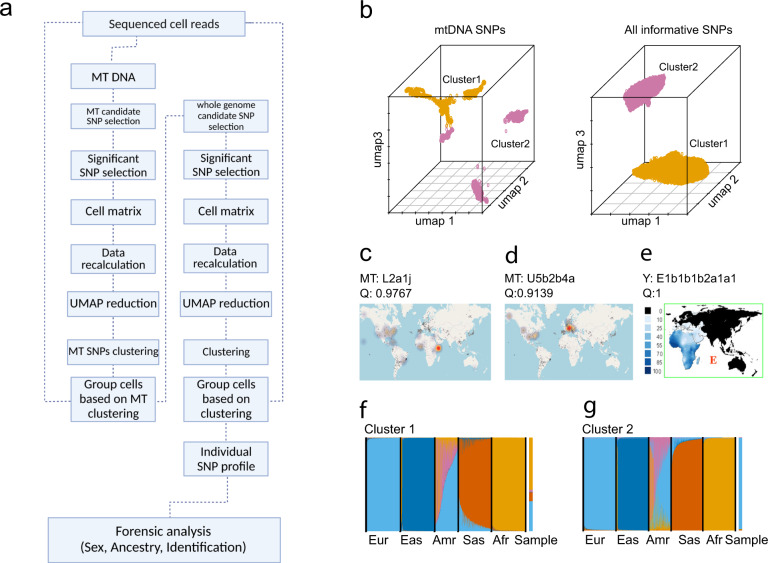
De-goulash bioinformatics pipeline for genetically deconvoluting a multi-person biological mixture with subsequent genetic characterization and individual identification of the separated mixture contributors based on single-cell transcriptome sequencing data. Pipeline description and application on balanced two-person blood mixture. **a** The de-goulash pipeline workflow for single-cell-based mixture deconvolution with pre-processing of the scRNA-seq sequencing data in two iteration steps (mtDNA SNP-based separation followed by genome-wide SNP-based separation). **b** The 3D UMAP representation of the two-step single-cell separation process of a balanced two-person blood mixture (dataset M2) involving one male contributor of East African ancestry and the one female contributor of European ancestry. **c** EMPOP^47^ map of the worldwide distribution of mtDNA haplogroup L2a1j inferred from haplogroup-diagnostic mtDNA SNPs of cell cluster 1 with inferred African maternal ancestry. **d** EMPOP map of mtDNA haplogroup U5b2b4a inferred from haplogroup-diagnostic mtDNA SNPs of cluster 2 with inferred European maternal ancestry. **e** Literature map^53^ of Y haplogroup E inferred from haplogroup-diagnostic Y-SNPs of cell cluster 1 with inferred African paternal ancestry. Cluster 2 did not present a Y haplogroup due to female sex, as also revealed in the genetic sex analysis for cluster 2, while for cluster 1 male sex was obtained. **f**, **g** Biparental ancestry analysis with STRUCTURE of the genome-wide SNPs obtained per each of the cell clusters with continental reference population data (Eur: Europeans, Eas: East Asians, Amr: Native Americans, Afr: Sub-Saharan-Africans), the result for the cells clusters are denoted as Sample, result for cell cluster 1 demonstrates inferred admixed biparental ancestry with a major African ancestry, result for cell cluster 2 demonstrates European biparental ancestry. The maternal, paternal, and bi-parental genetic ancestries inferred from cell cluster 1 and 2 agree with the family-based ancestries of the two individuals involved in the mixture.

Aiming to genetically separate, characterize, and individually identify persons who contributed to multi-person blood mixtures from single-cell transcriptome sequencing (scRNA-seq) data, we have developed a bioinformatics pipeline called *de-goulash* (Fig. 1a)^24^. We applied *de-goulash* on scRNA-seq datasets that we *de-novo* generated from multi-person blood mixtures and on in silico mixtures that we created by mixing publicly available single-person scRNA-seq datasets. Although there are several bioinformatic tools available for the separation of scRNA-seq data, such as ScSplit^25^, Souporcell^26^, or Vireo^27^, none of them allow streamlined application to combine single-cell separation with genetic characterization and individual genetic identification of the separated mixture contributors. *De-goulash* first deconvolutes mixtures, i.e., separates individuals who contributed to the mixtures in a two-step approach, using two sets of SNPs automatically called from the scRNA-seq data. The deconvoluted cell clusters, which correspond to the individuals who contributed to the mixture as described below, are then used to automatically call additional SNP sets per each of the separated cell clusters for genetic characterization regarding sex, biogeographic ancestry and individual genetic identification of the separated mixture contributors.

With de-goulash, after the alignment of the scRNA-seq data, two subsequent rounds of genetic mixture deconvolutions are applied. In the first iteration step, SNPs from mitochondrial DNA (mtDNA) that are abundant in the scRNA-seq data are called and used. Since mtDNA in humans is inherited uniparentally via the maternal line, multi-allelic mtDNA SNPs are caused by the presence of DNA from multiple individuals, with the rare exception of heteroplasmic sites in mtDNA. Therefore, mtDNA SNPs are suitable for mixture deconvolution given the many mtDNA SNPs with differences between individuals belonging to different maternal lineages (called mtDNA haplogroups). This first iteration allows a fast computation with less resources, since only a small subset of the large scRNA-seq data (i.e., the mtDNA part) is processed. Informative mtDNA SNPs are selected based on frequency across cells. To overcome the inherent problem of missing data in scRNA-seq, which creates gaps in positions in the SNP cell matrix, we applied the computational method DINEOF^28^. After recalculating missing data, the resulting cell-matrix is used for cluster analysis. Uniform manifold approximation and projection (UMAP)^29^ is applied for dimension reduction and visualisation. When the number of individuals in the mixture is unknown (or presumed unknown), we first determinedthe number of clusters using NbClust^30^, a collection of multiple clustering methods reaching a consensus about the ideal number of obtained cell clusters. The resulting matrix was used for K-means clustering, using a priori determined k or k obtained from NbClust calculation. In the second iteration step, de-goulash uses the cell clusters established based on mtDNA from the first iteration, to call suitable genome-wide SNPs per generated cell cluster. After filtering for informative SNPs and recalculating missing data, this expanded SNP list is used for the second clustering iteration that follows the similar steps as the first iteration.

After this two-step procedure, the pipeline uses the finally obtained cell clusters to automatically generate additional SNP sets per each of the separated cell clusters. These different sets of SNPs, selected based on different principles, are subsequently applied by the pipeline to characterize the separated mixture contributors regarding their sex and biogeographic ancestry (by use of population reference databases), and finally to individually identify the separated mixture contributors by use of a whole-exome sequencing reference database. In subsequent analyses, we also use the scRNA-seq data to obtain information about the tissue(s) of origin of the cells in the analysed mixture (using differentially expressed genes in each single-cell expression data clusters).

### Mixture deconvolution and genetic characterization from simple balanced mixtures

To test our approach, we first de novo generated scRNA-seq data from a simple two-person balanced blood mixture (dataset M2, Supplementary data 1), where the contribution of the two individuals was equal. For simplicity, the two individuals were selected to have different sex and different continental biogeographical ancestry (African and European). De-goulash revealed a clear separation of the cells in the mixture into two clusters in both iterations (Fig. 1b). In the first iteration, 62 mtDNA SNPs were used and separated 21.3% of the cells in the mixture, while in the second iteration 630 genome-wide SNPs were applied and separated nearly all of the cells (97%) (Supplementary Table 1).

To test if the two obtained cell clusters correspond to the two contributing individuals, we first performed genetic characterization analysis regarding sex and biogeographic ancestry for each of the two cell clusters separately (for individual genetic identification analysis, see below). To genetically determine sex, we first performed Y-chromosome SNP analysis and found very low number of Y-SNP sequencing reads for cluster 2, which we attributed to noise or errors in alignment, while for cluster 1 we detected ~10-fold more sequencing reads (Supplementary Fig. 1, Supplementary data 2). Second, we looked at the expression level of the gene coding for the non-coding RNA *XIST*, which is specifically expressed in somatic cells of biological females to inactivate one of the two X chromosomes^31^. After extracting the sequencing reads that map to the *XIST* gene, we plotted the expression level and found ~10-fold higher expression for cluster 2, and almost no expression for cluster 1 (Supplementary data 3, Supplementary Fig. 2). These results together allowed us to conclude that cell cluster 1 corresponds to a male and cluster 2 to a female, which agrees with the a priori knowledge about one female and one male in the blood mixture sequenced.

Genetic inference of biogeographic ancestry based on the two cells clusters was performed separately in three different ways using three different parts of the human genome allowing us to conclude biogeographic ancestry on three different levels. First, we established maternal ancestry, i.e., the person’s ancestry from the maternal side, by inferring the mtDNA haplogroups from the obtained mtDNA SNP data using Haplogrep2^32^, and investigated the geographic distribution of the identified mtDNA-haplogroups using literature knowledge. Here we found that cluster 1 (Fig. 1b) was assigned to mtDNA haplogroup L2a1j, which is most commonly observed in Africa (Fig. 1c), while cluster 2 was assigned to mtDNA haplogroup U5b2b4a, which is most commonly found in Europe (Fig. 1d). Both of these assignments were done with high confidence (*Q* = 0.9767 and 0.9139 respectively).

Second, we established paternal ancestry, i.e., a male’s ancestry from the paternal side, by inferring the Y-chromosomal haplogroups from the obtained Y chromosomal SNP data using Yleaf^33^, and investigated the geographic distribution of the identified Y-haplogroups using literature knowledge. For cell cluster 1, we detected Y-haplogroup E1b1b1b2a1a1, which shows a spatial distribution covering the Middle East and South Africa (Fig. 1e), while for cluster 2, no reliable Y-chromosomal data were obtained (Supplementary data 4) in agreement with the concluded female sex of the contributor of cluster 2.

Third, we inferred bi-parental biogeographic ancestry, i.e., a person’s ancestry from both paternal and maternal side, based on genome-wide autosomal SNPs using STRUCTURE^34^ and reference population data from the public 1000 Genomes Project^35^. To this end, per each cell cluster, genome-wide SNPs were filtered to be suitable for ancestry inference based on minor allele frequency difference between continental populations (max 0.3), and physical distance (min 500 kb) to adjust for linkage disequilibrium. For cell cluster 1, we obtained 53.6% African and 44.6% European ancestry, while other continental ancestries were minor (0.4% Native American, 0.2% South Asian), or zero (East Asian) (Fig. 1f, Supplementary data 5). For cluster 2, we revealed an almost complete (99.2%) clustering towards the European ancestry (Fig. 1g, Supplementary data 5).

Taken together, and supported by each of the three separate genetic ancestry analyses, our data allow us to conclude that the male individual of cluster 1 is of mostly African ancestry and the female individual of cluster 2 is of European ancestry. This genetic finding agrees with the a priori knowledge about the European female and African male in the sequenced blood mixture. Notably, based on a questionnaire, the male contributor originates paternally from East Africa. East Africa is not well represented in the 1000 Genomes reference data used (most African individuals are from Sub-Saharan Africa), which explains the higher African and lower non-African ancestry components we detected.

Furthermore, we determined the tissue of origin of the cells present in the mixture using gene expression profile derived from the same scRNA-seq data. Differentially expressed genes in each of the clusters obtained using t-SNE clustering analysis were used to determine the tissue and cell types via gene enrichment analysis with Enrichr (Human Gene Atlas)^36^. We found that the cell types in both clusters belong to different blood cell types, which is in agreement with the a priori knowledge that the scRNA-seq was generated from a blood mixture (Supplementary Fig. 3a).

**Fig. 2:**
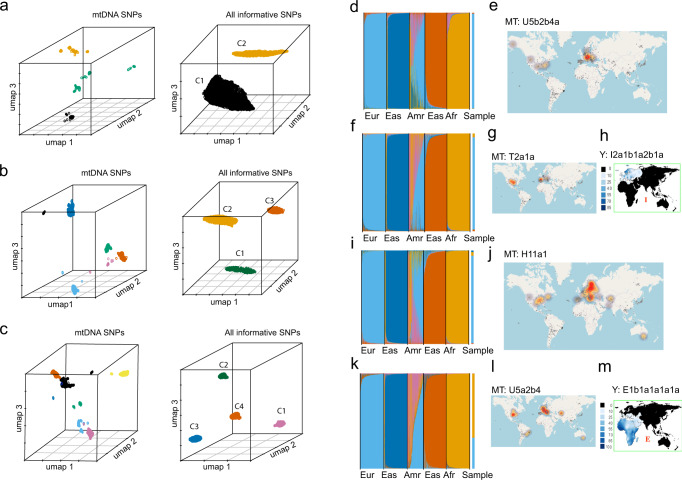
Mixture deconvolution and subsequent genetic characterization of the separated mixture contributors via de-goulash analysis of single-cell transcriptome sequencing data obtained from balanced blood mixtures involving two, three, and four individual contributors, respectively. **a**–**c** 3D UMAP representation of the single-cell separation of balanced blood mixtures involving two individuals (top, dataset M2-cl), three individuals (middle, dataset M3), and four individuals (bottom, dataset M4), respectively. The left panels show the results after the first iteration step of mixture deconvolution based on mtDNA SNPs. The right panels show the results after the second iteration based on genome-wide SNPs. **a** mixture of two European individuals with closely related mtDNA haplogroups separated in two distinct clusters after both iteration steps, **b** mixture of three European individuals separated in three distinct clusters after both iterations, **c** mixture of four individuals of diverse origin (individual 1&2: female, European ancestry, individual 3: male, European ancestry, individual 4: male, maternal European ancestry, paternal African ancestry) separated in 4 distinct clusters after both iterations. **d**, **f**, **i**, **k** Biparental ancestry analysis with STRUCTURE from autosomal SNPs obtained individual clusters from the 4-person mixture (Fig. 2c) with continental reference population data from 1000 Genomes Project data (Eur: Europeans, Eas: East Asians, Amr: Native Americans, Afr: Sub-Saharan-Africans) for **d** cell cluster 1 with inferred European biparental ancestry, **f** cell cluster 2 with inferred European ancestry, **i** cell cluster 3 with inferred European ancestry, and **k** cell cluster 4 with inferred major African ancestry. **e**, **g**, **j**, **l** EMPOP map of mtDNA haplogroups inferred from mtDNA SNPs from indiviudal clusters from the 4-person mixture in (Fig. 2c) for **e** cell cluster 1 with mtDNA haplogroup U5b2b4a (European maternal ancestry), **g** cell cluster 2 with mtDNA haplogroup T2a1a (European maternal ancestry), **j** cell cluster 3 with mtDNA haplogroup H11a1 (European ancestry), and **l** cell cluster 4 with mtDNA haplogroup U5a2b4 (European ancestry). **h**–**m** Literature maps^53^ of Y haplogroups inferred from haplogroup-diagnostic Y-SNPs per two of the four cell clusters from the 4-person mixture (Fig. 2C) for **f** cell cluster 2 with Y-haplogroup I2a1b1a2b1a (South-European ancestry), and **m** for cell cluster 4 with Y-haplogroup E1b1a1a1a1a (African ancestry).

Since the first iteration step of the mixture deconvolution procedure is solely based on mtDNA SNPs, one may speculate that the single-cell separation success is influenced by the degree of mtDNA differences between the individuals in the mixture. In order to test the impact of more closely related mtDNA haplogroups on the mixture deconvolution, we generated scRNA-seq data (dataset M2-cl, Supplementary data 1) from a second 2-person balanced blood mixture involving the individual 2 described above (European female with haplogroup U5b2b4a) and a new individual 3 (male with mtDNA haplogroup U5a2b4 of maternal European ancestry but with African paternal ancestry). Although, for technical reasons, the overall sequencing depth obtained from this blood mixture was relatively low (Supplementary data 1), a clear separation of cells into two clusters was revealed (Fig. 2a). While in the first iteration, three clusters were detected, which may be caused by the reduced number of mtDNA SNPs available due to the low sequencing depth and overall noise of the data, the second iteration demonstrated two clearly separated cell clusters, as expected for this two-person mixture. The results from the biological sex and biogeographic ancestry analysis agreed with the expectations from the a priori knowledge about the two individuals who contributed to this blood mixture (Supplementary data 3–5, Supplementary Figs. 2–4). These findings suggest that the degree of mtDNA differentiation of the to be separated individuals in a mixture does not negatively impact the success of our mixture deconvolution approach.

### Mixture deconvolution and genetic characterization from complex balanced mixtures

To further test our approach on more complex mixtures, we performed scRNA-seq on blood mixtures from more than two individuals. First, we generated a 3-person balanced blood mixture from three individuals of the same continental ancestry, all being Europeans, and performed scRNA-seq on this mixture with subsequent de-goulash data analysis (dataset M3, Supplementary data 1). While in the mixture deconvolution, the first iteration step did not provide a clear clustering, three distinct clusters were obtained after the second iteration (Fig. 2b) based on the vast majority (96.5%) of the cells (Supplementary Table 1) in agreement with the 3-person mixture. Genetic characterization analysis from the three separated cell clusters provided high confidence information about the sex, mitochondrial and Y-DNA haplogroups (Supplementary data 4) with inferred maternal and paternal ancestry, and biparental ancestry based on genome-wide SNPs (Supplementary Fig. 4, Supplementary data 5), which were in full agreement with the a priori knowledge on the presence two European females and one European male in this 3-person blood mixture.

Second, we produced a 4-person balanced blood mixture using the aforementioned three Europeans plus one African male and performed scRNA-seq and de-goulash data analysis (dataset M4, Supplementary data 1). As with the 3-person mixture, the first iteration of the mixture deconvolution did not provide clear separation (Supplementary Table 1, Fig. 2c), while the second iteration showed four distinct clusters using almost all (98%) of the cells (Fig. 2c) in agreement with the 4-person mixture. Genetic characterization analysis demonstrated the sex, haplogroups, and paternal, maternal and bi-parental biogeographic ancestries as expected from the a priori knowledge of the individuals in this 4-person blood mixture (Fig. 2d–m, Supplementary Figs. 1–4, Supplementary data 2–5).

**Fig. 3:**
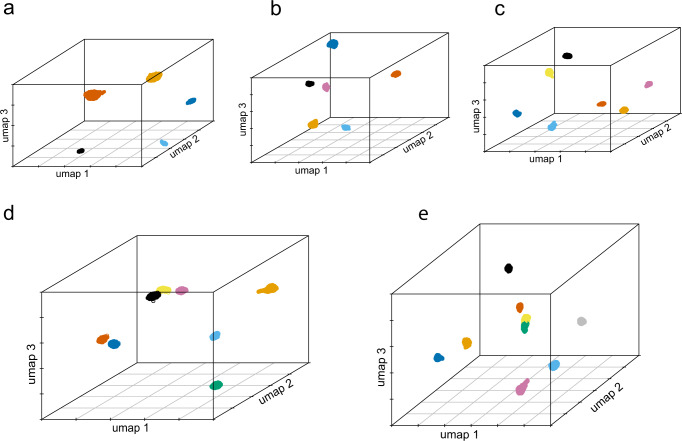
Deconvolution of balanced in silico mixtures involving 5–9 individual contributors via de-goulash analysis of single-cell transcriptome sequencing data. **a** Mixture of five individuals, **b** mixture of six individuals, **c** mixture of seven individuals, **d** mixture of eight individuals, and **e** mixture of nine individuals. Colour codes denote the different individuals from the respective mixtures. De-goulash uses a two-step approach with first iteration based on mtDNA SNPs and second iteration based on genome-wide SNPs.

Third, we generated in silico balanced mixtures containing 5–9 individuals per each mixture (datasets M5–M9). The 5-person in silico mixture was created by combining datasets M2 and M4 (one individual participated in both experiments therefore was present in both M2 and M4 dataset). Different in silico mixtures containing 6–9 individuals were created by combining four publicly available single individual scRNA-seq datasets with the M4 dataset (Supplementary Table 2). With de-goulash, for all of these in silico mixtures, we obtained the respective number of cell clusters that matched the number of individuals in the mixtures (Fig. 3a–e, Supplementary Fig. 5), including for the most complex 9-person mixture (Fig. 3e, Supplementary Fig. 5e). The separated cell clusters also revealed the correct information on sex, mtDNA and Y haplogroups and consequent maternal and paternal ancestry (Supplementary data 2–4 and Supplementary Table 3) as we deduced by analysing the individual datasets separately. These results suggest that with nine individuals representing the most complex mixture we tested, the limits of our mixture deconvolution approach have not been reached, and it is expected that balanced mixtures of more than nine individuals can be deconvoluted successfully with our approach. While maternal and paternal ancestry were derived correctly for all contributors in these mixtures, inferring biparental ancestry in individuals with more complex ancestry (datasets A1 and A2 in the in silico mixtures M6-M9, Supplementary Table 4) seems less reliable in the highly complex mixtures (Supplementary Fig. 4, Supplementary data 5), requiring further investigations.

### Individual genetic identification from deconvoluted balanced mixtures

Next, we investigated if individual genetic identification of the separated contributors is feasible based on the successfully deconvoluted scRNA-seq data obtained from the mixtures. For this purpose, we additionally generated whole-exome sequencing (WES) data from buccal swab reference samples of all individuals who contributed for the aforementioned blood mixtures, which served as study reference database for individual identification based on comparative matching (Supplementary Table 5). Autosomal SNPs were extracted from the scRNA-seq data from each of the deconvoluted cell clusters in all of the mixtures by taking two general criteria for identity SNP selection into consideration: (i) minimal difference in minor allele frequencies between the major population groups using the 1000 Genomes Project data with frequency not larger than 0.3, and (ii) the physical distance between the SNPs being larger than 500 kb to mitigate effects caused by linkage disequilibrium. Individual genetic identification was performed by matching identity SNPs obtained from each of the separated cell clusters in each of the mixtures against the WES reference database. Per each separated cell cluster and across mixtures, identity SNPs obtained from the cell clusters that overlapped with WES reference, thereby being used for genetic matching, ranged from 35 to 162 between clusters and mixtures.

**Fig. 4:**
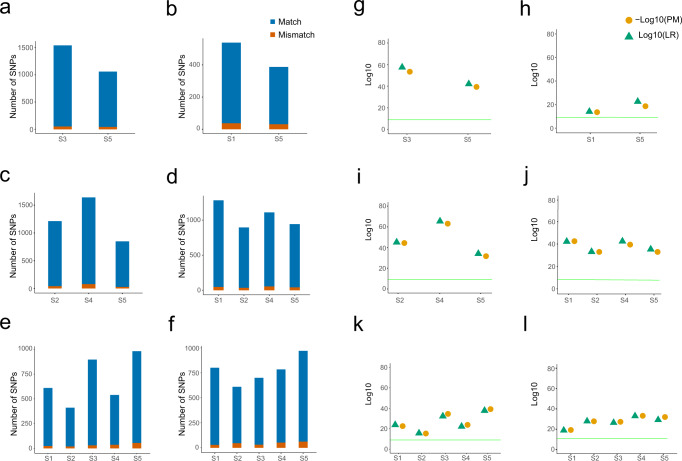
Individual genetic identification of separated mixture contributors via de-goulash analysis of single-cell transcriptome sequencing data obtained from balanced mixtures involving 2–9 individuals. Number of matching identity SNPs towards the whole-exome sequencing reference (**a**–**f**). **a** Blood mixture of two individuals (dataset M2), **b** blood mixture of two individuals with closely related mtDNA haplogroups (dataset M2-cl), **c** blood mixture of three individuals (dataset M3), **d** blood mixture of four individuals (dataset M4), **e** in silico data mixture of five individuals (dataset M5), **f** in silico data mixture of nine individuals of which only five individuals had WES reference data available for individual genetic identification (dataset M9). Statistical-based individual genetic identification. as logarithmic expression of LR and PM (**g**–**l**). Likelihood ratio (LR) and probability matching (PM) were used as statistical parameters to determine the strength of the evidence of a genetic match for individual genetic identification. Green line represents the 10E9 LR threshold for correct identification. **g** Blood mixture of two individuals (dataset M2), **h** blood mixture of two individuals with closely related mtDNA haplogroups (dataset M2-cl), **i** blood mixture of three individuals (dataset M3), **j** blood mixture of four individuals (dataset M4), **k** in silico data mixture of five individuals (dataset M5), **l** in silico data mixture of nine individuals of which only five individuals had WES reference data available for individual genetic identification (dataset M9).

To determine the strength of the evidence of a genetic match for individual genetic identification, likelihood ratio (LR) and probability matching (PM) were applied as statistical parameters. LR is used to determine whether the matching sample and reference sample came from the same individual^37^, while PM indicates the probability that the match has been caused by an unrelated individual. In a genetic identification process, generally a LR of more than 10E + 6 is considered as extremely strong evidence supporting the hypothesis in favour of individual identification^38^. Here, we used a more conservative 10E + 9 threshold since we are using a new technique. In all our datasets, we found a significant match (over 90% of SNPs) towards one of the samples in the study reference database (Fig. 4a–f, Supplementary Fig. 6). We attribute the percentage of non-matching SNPs to errors in either sequencing or minor bleed-through between clusters (Supplementary data 6). For all obtained individual matches, the LR of the matching SNP profile was significantly higher than the threshold used, ranging from 1.71E + 14 to 5.00E + 65 across matching samples, providing robust evidence for successful identifying the individual contributors to the analysed mixture (Fig. 4g–l). Notably, even in the most complex mixtures tested that included up to 9 individuals, where the number of separated cells was significantly lower, individual genetic identification of all mixture contributors was achieved successfully (Fig. 4l, Supplementary data 7). Subsequent inspection of the matching individuals that were used in the mixtures and in the study reference database confirmed the correct individual identification in all cases.

### Mixture deconvolution, genetic characterization and individual identification from imbalanced mixtures

**Fig. 5:**
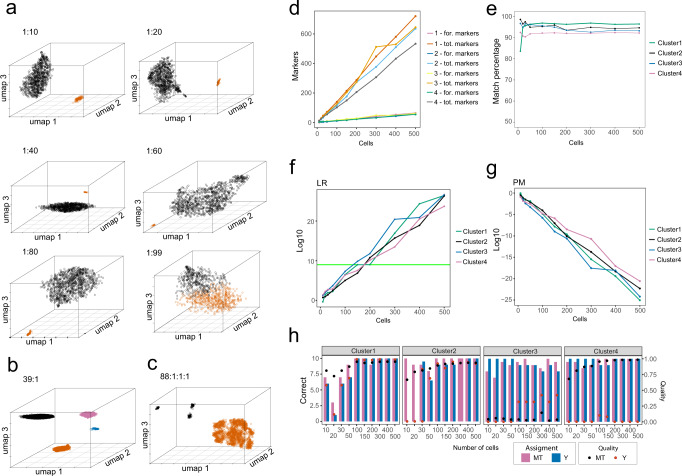
Analysis of imbalanced mixtures and testing of cell number limits in mixture deconvolution with subsequent genetic characterisation and individual genetic identification of the mixture contributors via de-goulash analysis of single-cell transcriptome sequencing data. **a** UMAP clustering showing the limitation of the single-cell separation process in imbalanced 2-person mixtures of various mixture ratios from 1:10 to 1:99. **b**, **c** UMAP clustering from single-cell mixture deconvolution of imbalanced 4-person mixtures with minor component of 3% of the total cell counts, **b** mixture with one minor component (blue), **c** mixture with three minor components (black). **d** Average number of identity SNPs used for individual identification (for. markers) and total number of available SNPs (tot. markers) per cluster per number of cells of deconvoluted and sampled cluster of mixture of four individuals (dataset M4). **e** Percentage of identity SNP alleles matching between cell cluster and WES reference database per cell cluster per number of cells (from deconvoluted clusters of dataset M4). **f** Average logarithmic expression of likelihood ratios per cell cluster per number of cells (from deconvoluted clusters of dataset M4). **g** Average logarithmic expression of match probability per cell cluster per number of cells (from deconvoluted clusters of dataset M4). **h** Average correct assignment of Y and mtDNA haplogroups per cell cluster from deconvoluted clusters of dataset M4 (bars) with marked average quality of the prediction (as dots).

Next, we tested our approach on more challenging imbalanced mixtures, i.e., mixtures to which the different individuals contributed differently. We started with imbalanced 2-person mixtures for which we selected 1000 cells from two datasets (A2 and A4) and mixed them in different proportions ranging from 1:10 to 1:99 (Supplementary Table 6). The cells of the minor component were selected from highly informative cells, i.e., cells that contain the highest number of sequencing reads in the respective dataset. The cell barcodes that were retained during the selection of the cells allowed us to evaluate the success of the separation process by comparing it to the original dataset from the balanced mixture. For the 1:10, 1:20, and 1:40 imbalanced in silico mixtures, a clear cluster separation according to the two individuals in the mixes were observed (Fig. 5a) without any “bleed-through” between the clusters (Supplementary Table 7). In the 1:60 dataset, we observed two cell clusters with a minor number of five cells of the minor component incorrectly assigned to the cluster of the major component (Supplementary Table 7). In the 1:80 dataset, although the data was visibly separated into two distinct cell clusters, significant incorrect assignment and bleed-through between clusters was seen (Supplementary Table 7). Finally, with the 1:99 dataset, the pipeline did not reach any cluster separation of the cells (Fig. 5a). These results suggest that for imbalanced 2-person mixtures, our approach is able to correctly deconvolute the two individual contributors with contributions up to about 1:60, at least.

Next, we tested our approach on more complex imbalanced in silico mixtures with more than two individual contributors by generating two imbalanced cell mixtures of four individuals from the aforementioned M4 dataset. These mixtures were generated by using earlier clustering assignments of the M4 dataset (Fig. 2c). The first imbalanced 4-person mixture included one minor component accounting for 3% of the total cells in the mixture and three major components with equal parts together accounting for the remaining 97% cells. Our approach achieved successful mixture deconvolution by revealing a clear separation of the four individual clusters (Fig. 5b). When compared to the cluster assignment of the original balanced dataset, we observed minimal changes in the assignments of cells toward the minor component (3 cells), and 49 cells were incorrectly assigned between the major component clusters (Supplementary Table 7).

In the second imbalanced 4-person mixture, we reverted the compositions of the minor and major components in that each of the three minor components represented 3% of the total number of cells, while the one major component represented the remaining 91% cells. Again, we obtained a clear separation of the four individual clusters (Fig. 5c). And again, the difference in the minor clusters compared to those in the original balanced dataset was minimal (two of them contain 1 cell previously differently assigned), while the observed bleed-through from the minor components into the major component was 42 cells total. This analysis suggests that in 4-person imbalanced mixtures, the minor components representing 3% of the total number of cells can be successfully deconvoluted in addition to the major ones.

When performing genetic characterization analyses on the dataset we obtained the correct information on sex, mtDNA and Y haplogroups and consequent maternal and paternal ancestry both in the major and minor clusters (Supplementary Table 8, Supplementary Figs. 7, 8). Though regarding bi-parental ancestry, the STRUCTURE results of the minor-component clusters did not result in clear evidence, likely due to the limited number of autosomal SNPs available for this analysis (Supplementary Table 8, Supplementary Figs. 7, 8).

Finally, we investigated the sensitivity of our approach for individual genetic identification by using the previously separated M4 dataset involving four contributors (Fig. 2c) and created data points of different numbers of cells ranging from 10 to 500. For each cell cluster, we randomly picked cellular barcodes to simulate various numbers of cells in a dataset. Each sampling was repeated 10 times to correct for selection bias and the results of the analysis pipeline were averaged and plotted to determine the average number of cells required for successful determination of each parameter. Genetic matching toward the study reference database for individual identification reaches the 90% matching SNP threshold already with 10 cells for most of the cases. Individual genetic identification was more unstable with low number of cells, but stabilised when more than 50 cells were included (Fig. 5d, e, Supplementary Table 9). However, the percentage of matching SNPs stayed above 90% for all of our collected data points consisting of more than 20 cells (Fig. 5d, e, Supplementary Table 9). For LRs, we observed an overall linear trend, especially beyond 30 cells (Fig. 5f, g, Supplementary data 8) similar trend can be observed with determination of haplogroups (Supplementary data 9 and 10). All clusters exceeded the conservative LR threshold (10E + 9) with 100–200 cells. These analyses suggest that at the sequencing depth and coverage we used here, our approach is able to deliver individual genetic identification on separated imbalanced multi-person mixtures containing more than 150 cells per individual contributor. This minimum number of cells is expected to further decrease with increase in scRNA-seq read depth, which will lead to an increase of the total number of detectable SNPs and thus an increase of the number of identity SNP available for matching.

### Comparison of de-goulash with other mixture deconvolution tools

In the past few years, some single-cell sequencing data analysis pipelines that allow mixture deconvolution such as ScSplit^25^, Souporcell^26^, and Vireo^27^ have been developed. However, unlike our *de-goulash* pipeline, none of them includes genetic characterisation and individual genetic identification steps, which are vital for future forensic applications in addition to mixture deconvolution. As direct comparison of these existing pipelines with our integrated pipeline *de-goulash* is therefore not possible, we have compared the mixture deconvolution part of *de-goulash* with the deconvolution-only pipelines. When we tested with a mixture of two individuals, with 5000 number of cells, all tested tools (SoupOrCell, Vireo, ScSplit and our tool de-goulash) were able to deconvolute the two individuals; however, ScSplit failed to assign 45.16% of cells from donor-1 to any cluster (Supplementary Fig. 9a, b). *De-goulash* consumed the least amount of RAM compared to SoupOrCell and Vireo (Supplementary Fig. 10a, b). SoupOrCell and Vireo took shorter time compared to *de-goulash* (Supplementary Fig. 10a, b). ScSplit, took less time but more recourses (Supplementary Fig. 10a, b). When we increased the complexity of the mixture to 9 individuals, with a total of 10,000 cells, Vireo performed as good as *degoulash* (Supplementary Fig. 9c, e), while SoupOrCell showed a high rate of wrong cell assignments in multiple clusters (Supplementary Fig. 9d) and ScSplit failed to deconvolute with similar recourses. The resources needed (RAM and time) to deconvolute a mixture of nine individuals follow similar trend to that the amount of resources needed to deconvolute a mixture of two individuals (Supplementary Fig. 10c, d). We conclude that for the mixture deconvolution stage, *de-goulash* is an accurate mixture deconvolution pipeline that requires fewer recourses compared to other deconvolution-only tools. With the streamlined integration of the genetic characterisation and individual genetic identification parts, as well as the tissue identification part, which all other software tools are lacking, *de-goulash* provides unique opportunity for future forensic applications, where mixture deconvolution represents the first step followed by genetic characterization in cases with unknown suspects and individual genetic identification in cases with known suspects and where knowledge of the tissue type of the mixture is highly relevant too.

## Discussion

The separation of individuals who contributed to biological mixtures, and their subsequent genetic characterization and/or individual identification, is crucial in many areas, especially in forensic investigation. In order to solve the long-standing challenge of mixture deconvolution, we hypothesized that, provided the availability of a suitable method, genetic information present in single-cell transcriptome data will allow to (i) separate the individuals who contributed to the biological mixtures, (ii) characterise the separated contributors such as regarding sex and ancestry, (iii) individually identify the separated contributors, and (iv) determine the tissue of origin of the cells in the mixture. We tested our hypothesis by developing a new approach based on single-cell sequencing and a dedicated bioinformatics pipeline and tested it in various scRNA-seq datasets obtained from de novo generated and in silico multi-person mixtures, simple and complex as well as balanced and imbalanced ones. Our proof-of-principle study demonstrates the feasibility of genetically separating individuals who contributed to multi-person blood mixtures with different levels of complexity (simple and complex, balanced and imbalanced mixtures) and genetically characterizing and individually identifying the separated contributors to the mixture.

While most previous attempts on mixture deconvolution, particularly in forensics, aimed at separating the contributors based on mixed DNA profiles, with our novel approach, we separated the individual contributors prior to genetic characterization and genetic individualization analyses. Downstream genetic analyses were thus performed as single-source analyses, thereby avoiding the challenges of characterizing and identifying individuals from mixed DNA profiles. We demonstrate that our approach is able to accurately separate individual contributors to biological mixtures containing up to nine individuals. However, with this maximal number of nine individuals in a mixture that we tested here, we did not see any limitations in mixture deconvolution. This suggests that our approach has the potential to successfully deconvolute mixtures of more than nine individuals, depending on the obtained number of SNPs per individual cell cluster, which warrants future experimental testing. As we demonstrated, the design of our deconvolution approach by considering mtDNA SNPs as the first step is not hindered by the degree of mitogenome similarity, as mixtures from individuals with closely as well as distantly related mtDNA haplogroups were separated equally well. As also shown, our approach can separate individuals from balanced and imbalanced mixtures up to a 1:60 ratio at least, and from 150 cells at least. However, single-cell sequence analysis with higher depths than done here will increase the number of available SNPs, thereby allowing successful deconvolution and downstream genetic characterization and identification analyses from less cells, which requires future empirical confirmation.

That our approach can cope successfully with highly imbalanced mixture is especially interesting in the forensic context, where imbalanced mixtures are more commonly found at crime scenes than balanced ones. Our approach opens new opportunities for future applications such as in forensics either directly or with further modifications and developments. In its current state, our approach has the potential to get applied in violent crime cases where multi-person blood mixtures are available for analysis, and regarding both scenarios: whether a suspect is already known to the investigative authorities or remains unknown. In cases with unknown suspects, genetic characterization of a sample donor via forensic DNA phenotyping of sex and bio-geographic ancestry—as studied here—but additionally also appearance traits, is crucial, as this can allow finding the unknown suspect via focussed police investigation. In order to achieve this, the considered SNPs need to be included in population reference data used for ancestry inference and in statistical models used for appearance prediction. Due to the large number of autosomal SNPs with redundant information on continental ancestry, and the redundancy in mtDNA and Y-DNA SNPs to characterize mtDNA and Y haplogroups to infer maternal and paternal ancestry, it simply is a matter of obtaining enough SNPs from the deconvoluted cells, and not necessarily specific ones, and not necessarily the same SNPs across different individual cell clusters within and between mixtures. Because of this and the sufficient numbers of mtDNA, Y-DNA, and autosomal SNPs we obtained from the separated cell clusters, our approach allows successful inference of maternal, paternal, and bi-parental ancestry for the separated mixture contributors. However, this is expected to be more challenging when it comes to extending the genetic characterization to additionally include appearance prediction, which works based on specific SNPs used in the statistical prediction models. For this extension of genetic characterization in the context of forensic DNA phenotyping it would be beneficial to move from transcriptome to genome sequencing of the biological mixtures, which will deliver more SNPs and thus potentially also specific SNPs used in appearance prediction models.

In cases with known suspects, the crucial forensic outcome is individual genetic identification of the sample donor via comparative forensic DNA profiling. To achieve this, individuals, such as those who contributed to a biological mixture and separated via the deconvolution approach here, are matched against a reference dataset obtained from a reference DNA sample of the known case suspect or from previously convicted crime offenders stored in a forensic DNA database. We showed that our approach allows individual genetic identification of separated mixture contributors from balanced and imbalanced multi-person mixtures with the highest statistical standard, which was possible because sufficiently enough identity SNPs were obtained from the separated individual cell clusters, respectively. However, because identity SNPs are acquired from every separated individual cell cluster, our approach is not working with universal identity SNPs, i.e., the same identity SNPs across all individuals. As for ancestry SNPs, there also is redundancy in identity SNPs, albeit based on the opposite population genetic features used for SNP selection. Hence, what matters too for individual genetic identification is to obtain enough identity SNPs, and not necessarily specific ones. Because no universal identity SNPs are used, the requirement for the reference dataset is to include as many as possible SNPs, and thus as many as possible identity SNPs. This way, there is a good chance that whatever set of identity SNPs being obtained from a cell cluster after successful mixture deconvolution is mostly available in the reference dataset used and thus available for matching. In the present study, we solved this complexity issue by using WES data as reference dataset due to the expected overlap between SNPs present in WES data with those obtained from transcriptome sequencing performed on the mixtures. Whole transcriptome sequencing could also be used on the reference samples, which would increase the number of identity SNPs available for genetic matching. This would be especially interesting for mixtures where a small number of separable cells per minor or all contributors are involved. Moreover, in the future, our single-cell mixture deconvolution approach could be transferred to genome sequencing for both the mixtures and the reference samples, which is expected to further increase the number of SNPs available for individual genetic identification (in addition to genetic characterization).

We envision that our approach could also be applied to investigative genetic genealogy (IGG) or forensic genetic genealogy (FGG) whereby dense SNP data set are used to find relatives of the donor of a crime scene sample via public genetic databases^39^. The approach has gained increased attention in recent years due to the successful identification of several missing persons and perpetrators. Indeed, our approach could discern individual profiles in a mixture to create single profile SNP data sets, although in the current study too sparse to be used in an IGG settings. Genetic imputation could further augment the data to a level where it can be uploaded to public databases for subsequent genealogy searches^40^.

In a forensic casework application, for a given suspect known by the police in a specific case, it would be possible to generate transcriptome or exome or genome sequencing data from the suspect’s reference sample, serving as prerequisite to solve a mixture case with our approach. Unfortunately, in many cases, no suspects are known to the police and thus no reference samples are available for D/RNA sequence analysis, where our approach with its genetic characterization part can help finding the unknown suspect and present him/her to standard forensic STR profiling. It currently appears unrealistic, however, that for solving cases with unknown suspects, national forensic DNA databases will include transcriptome or genome sequencing data in the near future. Maybe this will change with further developments in D/RNA sequencing technologies provided sequencing costs to decrease.

A disadvantage especially regarding future forensic applications is that the 10X genomics scRNA-seq platform we used here, requires live cells for successful genetic separation, which consequently limits the application to biological mixtures containing living cells. For a broader forensic application, alternative single-cell platforms not requiring living cells or those that can work with fixed cells should be tested and developed in the future. In addition, for mixtures with very low number of cells of all or minor contributor(s), deeper sequencing with higher coverage of the transcriptome or genome will be necessary to increase the total number of SNPs so that enough SNPs for successful genetic separation, genetic characterization, and individual genetic identification of the mixture contributors are available, which should be tested empirically too.

To conclude, in this study, we have developed a novel approach for genetically separating, characterizing and individually identifying contributors to biological mixtures. Our approach is based on single-cell sequencing of the biological mixtures for genetically separating the cells per each of the individual contributors, so that the subsequent genetic characterization and individual genetic identification of the separated mixture contributors became a single source analysis. In this proof-of-principle study, we demonstrate the feasibility of our approach on simple and complex as well as balanced and imbalanced mixtures. Future work needs to show transferability to other types of biological mixtures than the blood mixtures used here. Notably, our bioinformatic pipeline de-goulash works with any type of sequence dataset from which SNPs can be extracted, thereby allowing to move from transcriptome to genome sequencing in the future. Such further development is expected to increase the number of extractable SNPs, which will benefit the deconvolution of mixtures with (minor) contributors of low number of cells and genetic characterization and individual genetic identification of the separated mixture contributors, and may also allow expanding genetic characterization analyses towards appearance prediction. Further work may eventually allow applying our approach to biological mixtures found at crime scenes and in biomedical research where mixture deconvolution is required, such as identifying contaminations in cultures of cells, tissue and organoids.

## Methods

### Blood sample preparation for single-cell sequencing

From each donor blood was collected into a 10 mL EDTA anticoagulant tube using venepuncture procedure by a trained phlebotomist. PBMC were isolated by density gradient using Lymphoprep^TM^ (Stemcell Technologies, #07851) protocol. In short, first the blood was transferred into 15 mL tubes and centrifuged. The plasma was then removed and the sample was resuspended in 1 volume of PBS with 2% FBS. Samples were then layered on LymphoprepTM and centrifugated. The PBMC layer was transferred into PBS with 2% FBS, washed twice and filtered through 40 µL cell strainer. Cell viability was assessed using Countess II cell counter. A balanced mixture of the donors was prepared by mixing equal number of cells from each individual, and the resulting cell suspension was diluted as recommended by 10X Genomics single-cell preparation guide.

### Library preparation and sequencing

Single-cell RNA sequencing libraries were generated by following the 10X Chromium Single-cell library preparation protocols. The mixture M2 and M2-cl scRNA-seq library was prepared following the 10X Chromium Single-cell 3’ Reagent Kits v3 protocol. Mixture M3 and M4 were prepared using the 10X Chromium Next GEM Single-cell 3’ Reagent Kits v3.1 (dual index). The libraries were sequenced on a Illumina Novaseq6000. The sequencing depth, reads per cell and number of sequenced cells per experiment are available in Supplementary data 1.

### Data processing

Sequencing reads were aligned to the human genome (GRCh38) with the STAR aligner that is part of the Cell Ranger 3.0.2 software (10X Genomics). On average, we obtained a 91.63% alignment rate to the GRCh38 genome (alignment information available in Supplementary data 1). Valid cells were called based on total UMI counts per barcode. Expression matrix based on barcode, UMI, gene annotation and gene expression were used for grouping and t-SNE clustering. Differential expression was calculated by using the difference between the mean expression among clusters and cluster of interest.

### Mixture deconvolution

#### SNP calling

In order to start the 2-step deconvolution process, the aligned scRNA-seq data (BAM file) was filtered using two criteria with subset-bam v1.1.0^41^ (i) reads containing of cellular barcodes and (ii) BAM file containing only mtDNA reads (needed for the first iteration only). The resulting BAM file was indexed and sorted by TAG using samtools v.1.9^42^ and split into individual cell BAM files with a custom made Pysam v0.15.4^43^ script based on the cellular barcodes. Variants were called (on the whole dataset BAM file) with parallel FreeBayes v1.3.1^44^ using parsing arguments “-iXu -C 2 -q 1”. The resulting vcf file (containing the SNPs) was further filtered by bcftools filter QUAL < 80 DP < 100 (QUAL, quality; DP depth). In each individual cell, the number of reads supporting each SNP was counted using samtools mpileup. Indels were excluded and frequency table of each base was calculated for every SNP. SNPs with two or more bases per position were considered as variants of interest. Next variants were further filtered base on abundance between cells. For a variant to be considered, it needed to be present in minimum 1% of cells.

#### Generation of cell matrix and clustering

To filter cells, the variants obtained in the previous steps were applied to count the number of SNP reads per cells (base call quality ≥90, & read coverage of variant per cell ≥2). The cells were then filtered to contain a minimum of 20 SNPs (10 for imbalanced mixture datasets or when the data quality was low). The resulting cell matrix was used to impute the missing data using Dineof^28^. The recalculated matrix was utilized for dimension reduction and plotting using UMAP^29^ with parameters n_neighbors = 300, min_dist =0, n_components = 3 (the n_neighbors has been lowered to 50 for imbalanced datasets with reduced number of cells). When needed, if the number of individuals (number of clusters) in the mixture is not known, the number of clusters was determined by NbClust^30^. The rusting matrix was used for k-means clustering and plotting. By applying these steps, the first iteration was completed by generating a clustering assignment of cells based on mtDNA.

#### Cluster variant calling

To expand the SNPs from mtDNA and increase the cell number as well as effectivity of clustering, we first merged the cell BAM files based on the mtDNA clustering. After merging, variants were called using parallel FreeBayes v1.3.1^44^ with arguments “-iXu -C 2 -q 1”. The cluster variant lists were merged using Picard Tools version 2.25.6 MergeVcfs. The resulting vcf was filtered using bcftools filter (QUAL < 80 DP < 100) and non-unique variants were discarded with bcftools norm^45^. The created list was used to create counts per variant per cell and start a 2nd iteration to call SNPs and cluster cells (Fig. 1a). The BAM file and SNPs per cluster generated at the end of the 2nd iteration was used for the final analysis (biogeographical ancestry, sex, and individual identification).

### Whole-exome sequencing

Whole-exome sequencing (WES) was performed on DNA extracted from buccal swabs. Each individual was asked to rub their cheeks with a swab for 15 s on each side without touching their teeth. DNA was then extracted by adding 800 µl of water, 30 µ of Proteinase K (10 mg/ml), 90 µl of 10% SDS and incubated in 55 °C for 3 h. Next 300 µl of 5 M NaCl were added and the samples were incubated for 10 min at RT. After centrifugation the supernatant was mixed with 1 volume of isopropanol and centrifuged again. The pellet was then washed twice with 70% ethanol and dried. The resulting pellet was then dissolved in 50 µL of milliQ water and measured with pico green. Samples were then diluted to contain 500 ng of DNA in 30 µL. The quality of the DNA (integrity) was checked on 0.1% gel.

The library was prepared using a Hyperprep kit (Roche) with enzymatic fragmentation and dual index adapter ligation. Exome capture was performed using the SeqCap EZ MedExome probes (Roche). The samples were then sequenced on a Novaseq6000. The data was demultiplexed and high-quality reads were aligned to the human genome reference hg19 using the Burrow-Wheeler alignment tool (BWA version 0.7.3a). Base quality score was recalibrated and indels realigned using Genome Analysis ToolKit (GATK version 3.7)^46^. Duplicates were marked using Picard (Picard Tools version 1.90). Variant calling was performed with HaplotypeCaller (GATK v3.8). Subsequently, the samples were pooled for combined calling with GATKs GenotypeVCFs and VariantQualityScoreRecalibration workflow. Sample QC metrics were obtained using GATKs DepthOfCoverage and VariantEvaluation modules. Background noise levels were estimated and corrected for using the verifyBAMid tool and the “contamination fraction” option in GATKs HaplotypeCaller.

### Genetic characterisation analyses

#### Uniparental ancestry analysis

Maternal (mtDNA) ancestry was acquired by applying Haplogrep2.1.20 on vcf file of each cluster after the vcf file using bcftools filter (QUAL < 80 DP < 20). The results of the analysis were compared to an mtDNA database EMPOP^47^ (for geographical density of mtDNA haplogroup), and PhyloTree^48^ (for phylogenetic tree of mtDNA variations). The Y chromosome ancestry was determined using Y-leaf^33^ that uses the cluster BAM file as an input and parameters –b 90 –q 20 –r 2 as recommended by the user manual.

#### Sex determination

The presence of Y chromosome was determined by counting the number of reads that align to the Y chromosome and comparing between different clusters. The expression level of the long non coding RNA, XIST RNA, (from the X chromosome) that covers the inactive X chromosome in female cells, was used to determine the presence of an inactive X chromosome. The location of XIST gene was determined using Ensembl^49^ gene coordinates. The reads in the XIST gene and Y chromosome were extracted from the SAM file and counted using samtools^45^.

#### Bi-parental ancestry analysis

We first determined the match percentage by comparing the variants from each cluster (from scRNA-seq) and the exome reference. A match between a reference exome and a given cluster is called if the match percentage was more than 90%. For further processing, non-matching SNPs (SNPs with no match between the exome reference and the cluster) and only SNPs common between the exome reference and the cluster were retained. Variants were further filtered based on presence in the 1000 Genomes database^35^. Next, we generated a 1000 Genomes reference data set using the five continental populations (European, African, American, South Asian, and East Asian) in the 1000G project. A pruning step was performed to avoid linkage disequilibrium effects where a minimum of 500 kb distance was required between included SNPs. For each sample, biparental ancestry analysis was performed using the STRUCTURE (v2.3.4)^34^. Briefly, the software uses a statistical model to iteratively assign each individual to fractions of a number of presumed populations until the model is assumed to converge. We performed 10,000 burn-in s iterations and 10,000 subsequent iterations with five presumed populations (*K* = 5) with the admixture model was applied.

### Individual genetic identification analysis

The results from the biparental ancestry analysis were used to determine the dominant population in the sample (cluster), which was in turn used to extract allele frequency (AF) for our SNPs. For the calculations of forensic parameters, the SNPs were further pruned to only include genetic markers where the allele frequencies did not vary by more than 0.3 between the populations. Simultaneously we pruned the SNPs using a 500 kb distance between included markers, which mitigates potential effects of linkage disequilibrium. We then computed:

1. Total random match probability (RMP) using:
  $${{{{{\rm{RMP}}}}}}\,=\mathop{\prod }\limits_{i=1}^{N}\Pr ({G}_{i})=\Pr ({G}_{1})\Pr ({G}_{2})\ldots \Pr ({G}_{N})$$
  where *i* denotes the *i*’th SNP, *N* the total number of SNPs and Pr(*G*_*i*_) takes the value AF_i_^2^ for homozygous genotypes, *G*_*i*_, and 2AF_i_(1-AF_i_) for heterozygous genotypes and where AF_i_ is the allele frequency of the SNP. We have assumed Hardy Weinberg Equilibrium throughout our calculations although adjusting for disequilibrium is a small change.
2. The likelihood ratio (LR) was derived directly from the RMP as
  $${{{{{\rm{LR}}}}}}=\frac{1}{{{{{\rm{RMP}}}}}}$$
  where we have assumed the scenario where there is a perfect match between the sample’s genotype and some reference (not used in this study), whereas the model can easily be expanded to account for allelic dropouts/dropins and other errors.
3. Combined match probability (CPM), referred to only as PM in the text, was calculated as
  $${{{{{\rm{CPM}}}}}}=\mathop{\prod }\limits_{i=1}^{N}\mathop{\sum }\limits_{g=1}^{G}\Pr {({G}_{i,g})}^{2}$$
  where the inner summation traverses all possible genotypes (*G*_*i,g*_) at marker *i* and summarizes the probability to observe two identical genotypes at each marker. The CPM is the product of the probabilities for each marker.

Note that 1 and 2 are related to the specific DNA profile whereas 3 is related to the average statistics for the markers available from the cluster and remaining after pruning.

### Analysis of public scRNA-seq datasets

Four publicly available scRNA-seq datasets were obtained from 10x genomics (https://www.10xgenomics.com/resources/datasets). SNPs were called using FreeBayes v1.3.1^44^ parsing arguments “-iXu -C 2 -q 1–throw-away-indels-obs”. The SNP vcf file was filtered using bcftools filter QUAL < 80 DP < 20, and was used for further analysis. Maternal, paternal, and biparental ancestry were determined as described above.

### In silico mixtures to determine the impact of balanced and imbalanced mixtures

#### Generation and analysis of balanced mixtures

Balanced mixture containing between 5 and 9 individuals were generated by randomly selecting barcodes from each dataset (Supplementary Table 2 for all in-silico mixture contents) and merging the reads from the selected barcodes of each dataset. Each dataset retained the cellular barcode information to allow further assessment. The number of cells in each mixture is available in Supplementary Table 2. The datasets were processed via the deconvolution and analysis pipeline as described above.

#### Generation and analysis of imbalanced mixtures

Imbalanced mixtures were made using two datasets (A2, A4) that were obtained from publicly available sources by randomly selecting a total of 1000 cells. For the major dataset (A4) all available cells were used. For the minor dataset (A2) 1000 cells, with the most reads per cell, were preselected to avoid skewing of the analysis with low information cells. The ratio between the minor and major component ranged between 1:9 and 1:99. Each of the datasets was then filtered for the reads containing the selected barcodes. The subsets of the resulting dataset were merged into a new mixture. For the separation we used a modified deconvolution pipeline using lowered number of SNPs and UMAP neighbours to reflect lower number of cells. In this, due to the low number of cells, the SNP filtering parameters of QUAL < 50 DP < 50 were used. The data were further analysed using the analysis pipeline with the described modification for limited datasets as well as analysing the correct assignment of each cell to cluster of its original source.

#### Generation and analysis of imbalanced mixtures of higher degree

Based on the deconvolution cluster assignment of M4 dataset we randomly selected cell barcodes from each cluster. We then filtered the reads of the selected barcodes to create an imbalanced subset of the original dataset. Next, we generated two datasets, each containing proportional mix of minor and major components. In the first mixture, we selected one minor component (3% of total cells) and three major components (proportionally 97% of total cells). The second mixture contained three minor component cluster (each 3% of total cells) and one major component cluster (remaining 91% of total cells). The mixtures were processed using the deconvolution and analysis pipeline as previously described.

### Determination of cell number limitation

For each cluster of the M4 dataset, we randomly selected 10–500 cell barcodes (based on previous deconvolution and cluster assignment of cells). For every point (number of barcodes) we selected 10 times to correct for batch effect. Reads of the selected barcodes were filtered from the original M4 dataset creating a new subset dataset. Each subset dataset had variants called using FreeBayes v1.3.1^44^ with arguments “-iXu -C 2 -q 1–throw-away-indels-obs”. Next, the analysis pipeline was performed for each subset (using the called SNP vcf file and the subset BAM file as input). The results of exome match and forensic parameters per point were averaged. Results for haplogroup assignment were given 1 or 0 if the haplogroup was correct or incorrect, respectively. The value of 0.5 was given when the haplogroup was 1 branch up according to PhyloTree^48^. The accumulated scores were then averaged.

### Deconvolution pipeline comparison

In total four mixture deconvolution pipelines were tested (ScSplit 1.0.8, Vireo 0.2.3, SoupOrCell 2.0, De-goulash) on two in silico mixtures. The first silico mixture was prepared by mixing two single donor datasets (datasets A3 and A4, see Supplementary Table 2) that generated a total of 5000 cellular barcodes (2500 per donor). The respective bam files have been subsetted and merged using samtools 1.9. The second in silico mixture used for the comparison of the pipelines was a complex mixture that was generated by mixing de novo generated data and single donor datasets (M9 mixture, see Supplementary Table 2).

For each pipeline we followed the provided manual and applied the recommended parameters. Preprocessing for scSplit was done with samtools 1.9 for Vireo with cellSNP 0.3.1. Since Vireo and SoupOrCell require the number of individuals in the mixture to be known, we provided the number of individuals. For each pipeline, the amount of time consumed, the amount of recourses used, and the final clustering (deconvolution was recorded) were compared.

### Reporting summary

Further information on research design is available in the Nature Portfolio Reporting Summary linked to this article.

## Supplementary information

Supplementary Information

Description of Additional Supplementary Files

Supplementary Data 1

Supplementary Data 2

Supplementary Data 3

Supplementary Data 4

Supplementary Data 5

Supplementary Data 6

Supplementary Data 7

Supplementary Data 8

Supplementary Data 9

Supplementary Data 10

Reporting summary

## Data Availability

The individual datasets used in the in silico part of the study are available via the 10x website: A1: https://www.10xgenomics.com/resources/datasets/5-k-peripheral-blood-mononuclear-cells-pbm-cs-from-a-healthy-donor-v-3-chemistry-3.0.2 A2: https://www.10xgenomics.com/resources/datasets/peripheral-blood-mononuclear-cells-pbm-cs-from-a-healthy-donor-chromium-connect-channel-1-3.1.0 A3: https://www.10xgenomics.com/resources/datasets/4-k-pbm-cs-from-a-healthy-donor-2.1.0 A4: https://www.10xgenomics.com/resources/datasets/10-k-pbm-cs-from-a-healthy-donor-gene-expression-and-cell-surface-protein-3.0.0 The mixture datasets that were *de-novo* generated in this study are available at the EGA database with EGAS00001006202. The UMAP coordinate files and STRUCTURE 1000Genomes clustering used to generate the clustering graphs can be found on figshare^50–52^.

## Code availability

The bioinformatics pipeline de-goulash^24^ is available at: https://github.com/genid/de-goulash.

## References

[1] Kayser M, De Knijff P (2011). Improving human forensics through advances in genetics, genomics and molecular biology. Nat. Rev. Genet..

[2] Bennett L (2019). Mixture deconvolution by massively parallel sequencing of microhaplotypes. Int. J. Leg. Med..

[3] Holland MM, McQuillan MR, O’Hanlon KA (2011). Second generation sequencing allows for mtDNA mixture deconvolution and high resolution detection of heteroplasmy. Croat. Med. J..

[4] Perlin MW (2011). Validating TrueAllele (R) DNA Mixture Interpretation. J. Forensic Sci..

[5] Novroski NMM (2019). Expanding beyond the current core STR loci: an exploration of 73 STR markers with increased diversity for enhanced DNA mixture deconvolution. Forensic Sci. Int. Genet..

[6] Hwa HL (2018). A 1204-single nucleotide polymorphism and insertion-deletion polymorphism panel for massively parallel sequencing analysis of DNA mixtures. Forensic Sci. Int. Genet..

[7] Gill P, Jeffreys AJ, Werrett DJ (1985). Forensic application of DNA fingerprints. Nature.

[8] Vuichard S (2011). Differential DNA extraction of challenging simulated sexual-assault samples: a Swiss collaborative study. Investig. Genet..

[9] Kayser M (2017). Forensic use of Y-chromosome DNA: a general overview. Hum. Genet..

[10] Alladio E (2018). DNA mixtures interpretation - A proof-of-concept multi-software comparison highlighting different probabilistic methods’ performances on challenging samples. Forensic Sci. Int. Genet..

[11] Budowle B (2009). Mixture Interpretation: Defining the Relevant Features for Guidelines for the Assessment of Mixed DNA Profiles in Forensic Casework. J. Forensic Sci..

[12] Gill P (1998). Interpreting simple STR mixtures using allele peak areas. Forensic Sci. Int..

[13] Buckleton JS (2019). The Probabilistic Genotyping Software STRmix: Utility and Evidence for its Validity. J. Forensic Sci..

[14] Anslinger K, Bayer B (2019). Whose blood is it? Application of DEPArray (TM) technology for the identification of individual/s who contributed blood to a mixed stain. Int. J. Leg. Med..

[15] Williamson VR, Laris TM, Romano R, Marciano MA (2018). Enhanced DNA mixture deconvolution of sexual offense samples using the DEPArray system. Forensic Sci. Int. Genet..

[16] Anslinger K, Graw M, Bayer B (2019). Deconvolution of blood-blood mixtures using DEPArray(TM) separated single cell STR profiling. Rechtsmedizin.

[17] Elliott K, Hill DS, Lambert C, Burroughes TR, Gill P (2003). Use of laser microdissection greatly improves the recovery of DNA from sperm on microscope slides. Forensic Sci. Int..

[18] Fontana F (2017). Isolation and genetic analysis of pure cells from forensic biological mixtures: The precision of a digital approach. Forensic Sci. Int. Genet..

[19] Verdon TJ, Mitchell RJ, Chen W, Xiao K, Van Oorschot RAH (2015). FACS separation of non-compromised forensically relevant biological mixtures. Forensic Sci. Int. Genet..

[20] Watkins DRL, Myers D, Xavier HE, Marciano MA (2021). Revisiting single cell analysis in forensic science. Sci. Rep..

[21] Kayser M (2015). Forensic DNA Phenotyping: predicting human appearance from crime scene material for investigative purposes. Forensic Sci. Int. Genet..

[22] Phillips C (2015). Forensic genetic analysis of bio-geographical ancestry. Forensic Sci. Int. Genet..

[23] Tang X, Huang Y, Lei J, Luo H, Zhu X (2019). The single-cell sequencing: new developments and medical applications. Cell Biosci..

[24] Kulhankova, L. et al. De-goulash cell deconvolution and forensic analysis pipeline. 10.5281/zenodo.7559996 (Github, 2022).

[25] Xu J (2019). Genotype-free demultiplexing of pooled single-cell RNA-seq. Genome Biol..

[26] Heaton H (2020). Souporcell: robust clustering of single-cell RNA-seq data by genotype without reference genotypes. Nat. Methods.

[27] Huang Y, McCarthy DJ, Stegle O (2019). Vireo: Bayesian demultiplexing of pooled single-cell RNA-seq data without genotype reference. Genome Biol..

[28] Zheng, S., Huang, S. X. & Fang, H. X. *Data Filling from Incomplete Oceanographic Datasets Using EOF Calculations*. (World Acad Union-World Acad Press, 2008).

[29] McInnes, L., Healy, J. & Melville, J. UMAP: Uniform Manifold Approximation and Projection for Dimension Reduction. Preprint at *arXiv*10.48550/arXiv.1802.03426 (2020).

[30] Charrad M, Ghazzali N, Boiteau V, Niknafs A (2014). Nbclust: an R package for determining the relevant number of clusters in a data set. J. Stat. Softw..

[31] Pontier DB, Gribnau J (2011). Xist regulation and function eXplored. Hum. Genet..

[32] Weissensteiner H (2016). HaploGrep 2: mitochondrial haplogroup classification in the era of high-throughput sequencing. Nucleic Acids Res..

[33] Ralf A, Montiel Gonzalez D, Zhong K, Kayser M (2018). Yleaf: software for human Y-chromosomal haplogroup inference from next-generation sequencing data. Mol. Biol. Evol..

[34] Pritchard JK, Stephens M, Donnelly P (2000). Inference of population structure using multilocus genotype data. Genetics.

[35] 1000 Genomes Project Consortium. (2015). A global reference for human genetic variation. Nature.

[36] Chen EY (2013). Enrichr: interactive and collaborative HTML5 gene list enrichment analysis tool. BMC Bioinforma..

[37] Collins A, Morton NE (1994). Likelihood ratios for DNA identification. Proc. Natl Acad. Sci. USA.

[38] Martire KA, Kemp RI, Sayle M, Newell BR (2014). On the interpretation of likelihood ratios in forensic science evidence: presentation formats and the weak evidence effect. Forensic Sci. Int..

[39] Greytak EM, Moore C, Armentrout SL (2019). Genetic genealogy for cold case and active investigations. Forensic Sci. Int..

[40] Das, S., Abecasis, G. R. & Browning, B. L. in *Annual Review of Genomics and Human Genetics,* Vol. 19 (eds A. Chakravarti & E. D. Green) 73–96 (2018). 10.1146/annurev-genom-083117-021602 29799802

[41] Genomics, x. *subset-bam*, https://github.com/10XGenomics/subset-bam (2020).

[42] Danecek P (2021). Twelve years of SAMtools and BCFtools. GigaScience.

[43] pysam-developers. *Pysam*, https://github.com/pysam-developers/pysam (2020).

[44] Garrison, E. & Marth, G. Haplotype-based variant detection from short-read sequencing. Preprint at *arXiv*10.48550/arXiv.1207.3907 (2012).

[45] Li H (2009). The Sequence Alignment/Map format and SAMtools. Bioinformatics.

[46] McKenna A (2010). The Genome Analysis Toolkit: a MapReduce framework for analyzing next-generation DNA sequencing data. Genome Res..

[47] Parson W, Dür A (2007). EMPOP—A forensic mtDNA database. Forensic Sci. Int. Genet..

[48] van Oven M, Kayser M (2009). Updated comprehensive phylogenetic tree of global human mitochondrial DNA variation. Hum. Mutat..

[49] Howe KL (2021). Ensembl 2021. Nucleic Acids Res..

[50] Kulhankova, L. et al. Clustering files Iteration1, 10.6084/m9.figshare.21790061.v2, (Figshare, 2022).

[51] Kulhankova, L. et al. Clustering files Iteration2, 10.6084/m9.figshare.21790061.v2, (Figshare, 2022).

[52] Kulhankova, L. et al. STRUCTURE clustering files, 10.6084/m9.figshare.21792344.v2, (Figshare, 2022).

[53] Chiaroni J, Underhill PA, Cavalli-Sforza LL (2009). Y chromosome diversity, human expansion, drift, and cultural evolution. Proc. Natl Acad. Sci. USA.

