# Supplementary material for: Single-cell transcriptome sequencing allows genetic separation, characterization and identification of individuals in multi-person biological mixtures
Source: Commun Biol. 2023 Feb 20;6:201. doi: 10.1038/s42003-023-04557-z (PMC9941516; doi:10.1038/s42003-023-04557-z)
Supplement: Supplementary file 1 — Supplementary Information [file 42003_2023_4557_MOESM1_ESM.pdf]

Supplementary figure 1: The percentage of Y chromosomal reads per cluster compared to the total read count.

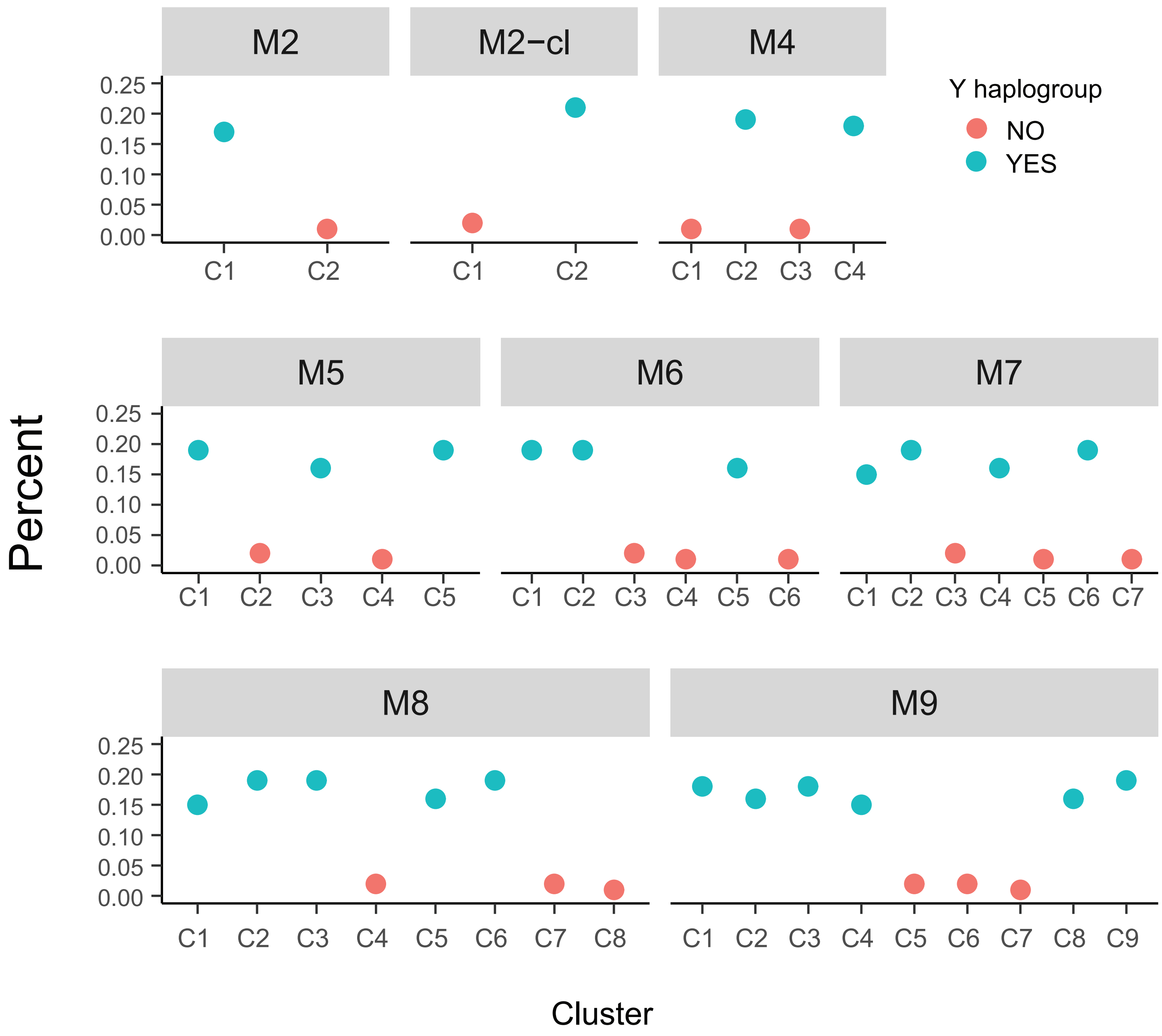

Each panel shows a deconvoluted mixture of different complexity ranging from 2-9 individuals. Higher expression suggest the cluster is male. In addition, colors in the graph indicate the assignment of Y chromosome haplogroup to each cluster.

Supplementary figure 2: The percentage of reads in XIST compared to the overall expression over the X chromosome.

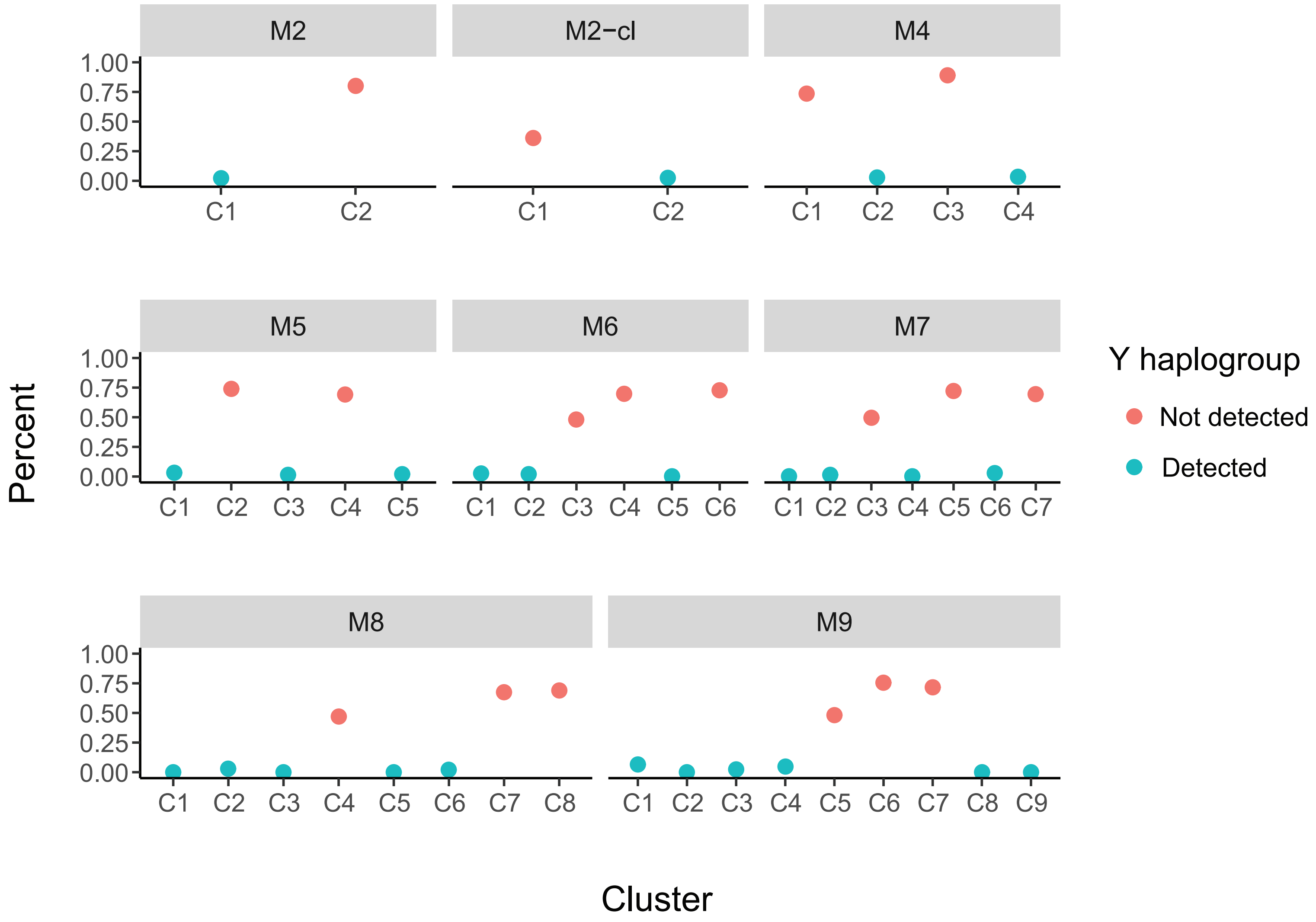

Each panel shows a deconvoluted mixture of different complexity from 2-9 individuals. Higher expression suggest the cluster is female. In addition, colours in the graph indicate the assignment of Y chromosome haplogroup to each cluster.

Supplementary figure 3: Evaluation of tissue of origin of biological mixtures.

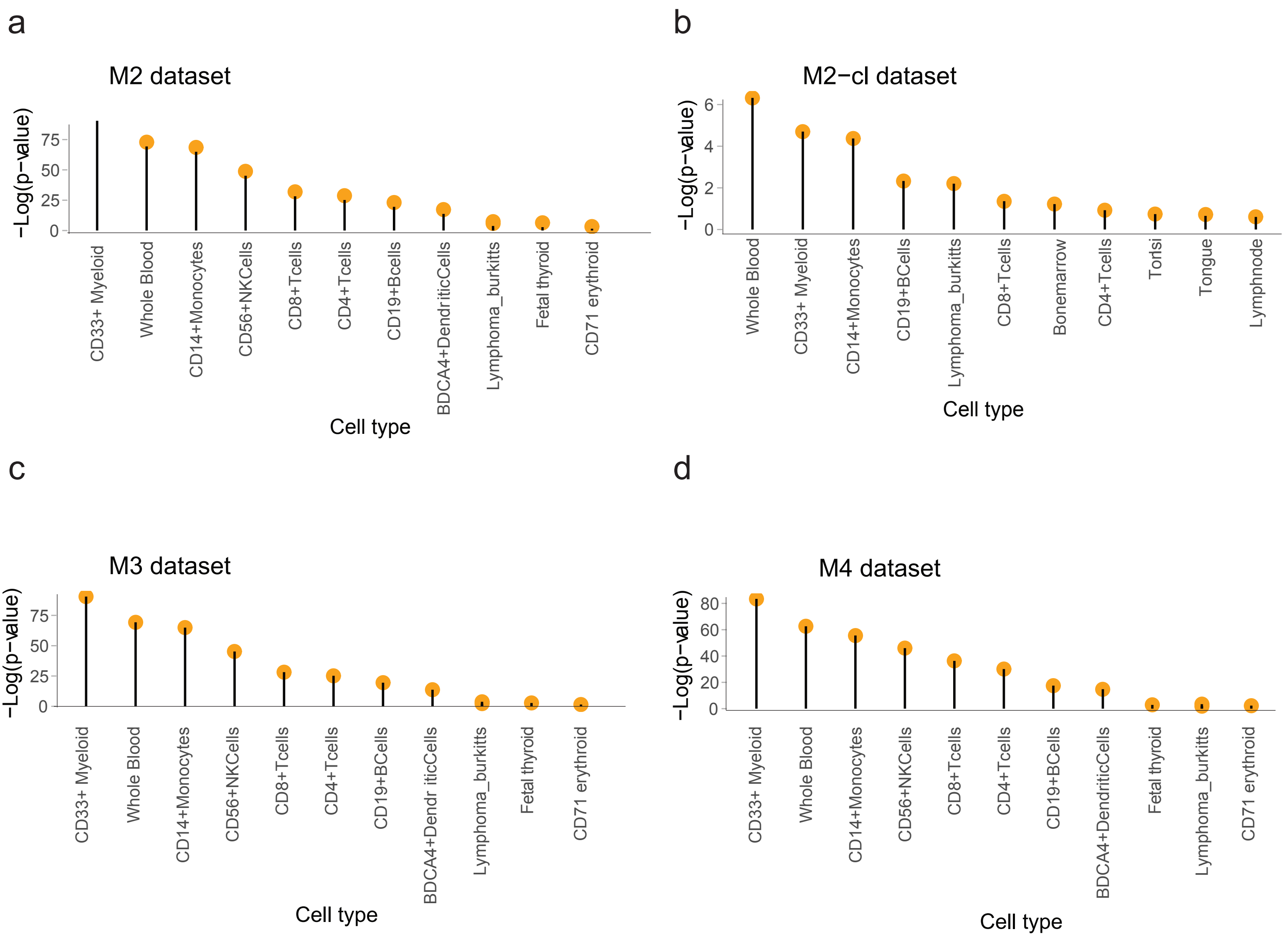

Pools of differentially expressed genes between clusters of t-SNE were assessed by Enricher to investigate the tissue of origin of the mixture. a) For biological mixture of two individuals . b) For biological mixture of 2 individuals with close mitochondrial ancestry. c) For mixture of 3 individuals with European ancestry. d) For mixture of 4 individuals with diverse background. As expected, the analysis indicate the tissue of origin of these mixtures is blood.

Supplementary figure 4: Biparental ancestry assessed using STRUCTURE after deconvolution of mixtures (M2-M9) and matching towards the reference

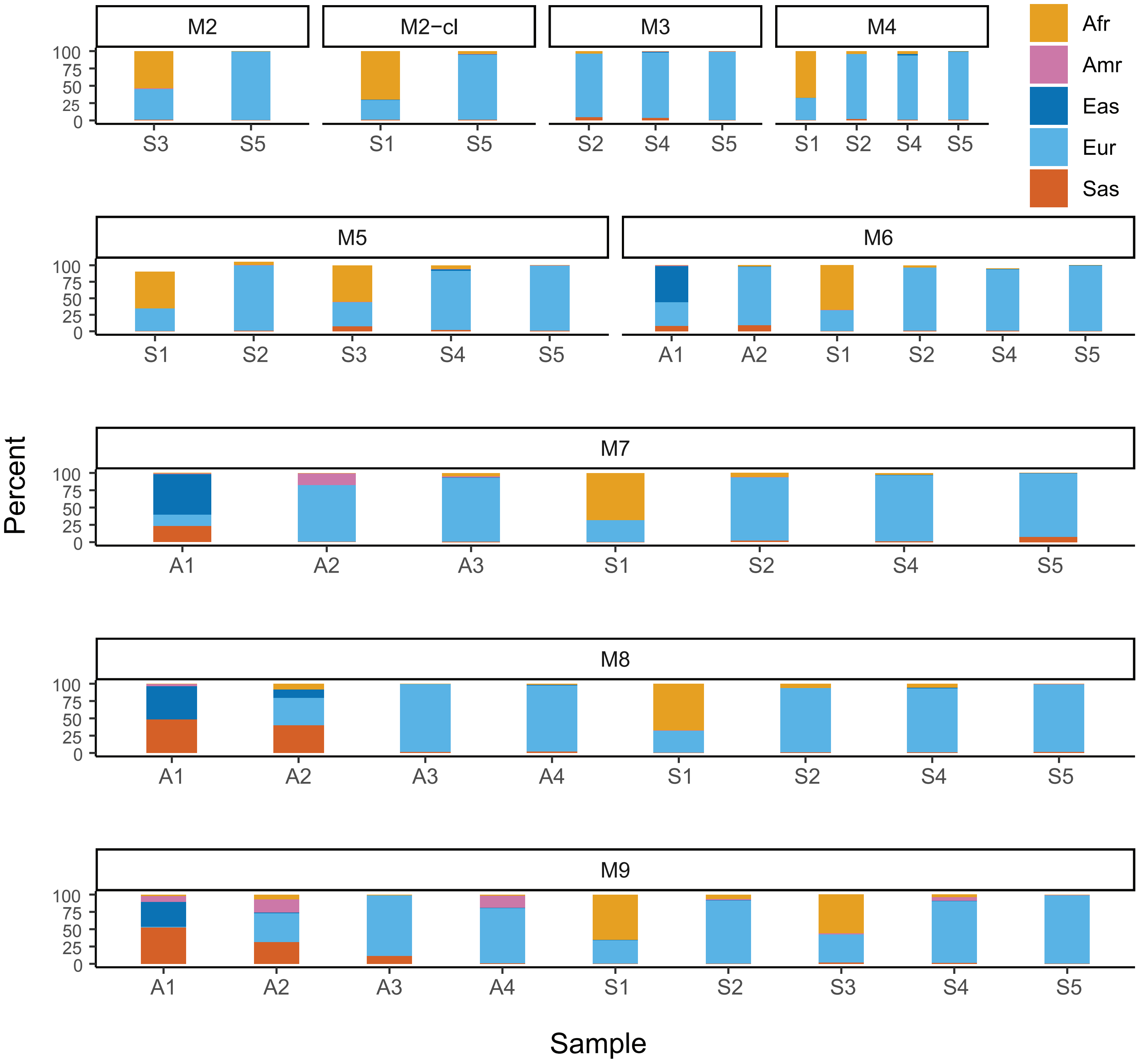

Each cluster was assessed separately with the available SNPs and compared to profiles gathered from 1000 Genomes database with known populations. Each panel represents a mixture and its clusters with regard to similarity of the profiles towards the assessed populations. The ancestry determination is stable between the different mixtures with the exception of datasets with complex ancestry (A1 and A2 datasets).

Supplementary figure 5: UMAP and K-means clustering of in silico mixtures in the first iteration using mtDNA SNPs.

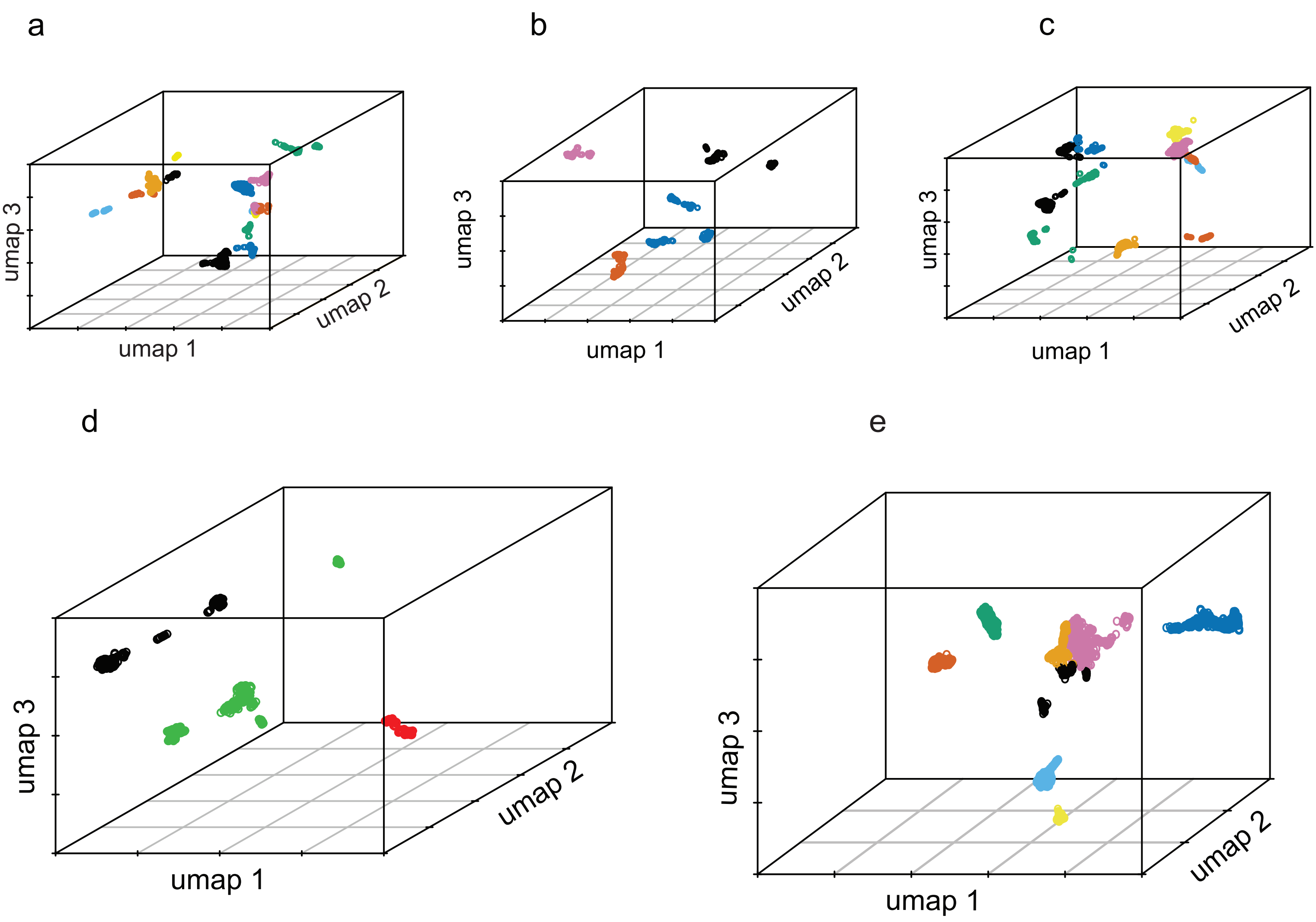

a) Mixture of 5 individuals, in the first iteration the clustering remained fairly unclear. **b)** Mixture of 6 individuals. NbClust detected a lower number of cluster with very unclear clustering result. **c)** Mixture of 7 individuals. NbClust detected 8 cluster with unclear borders. **d)** Mixture of 8 individuals NbClust detected a lower number of individual cluster. **e)** Mixture of 9 individuals. NbClust detected 8 clusters while some clusters appear clean and well separated, others cluster close together with little to separate them

Supplementary figure 6: Number of matching and non-matching identity SNPs

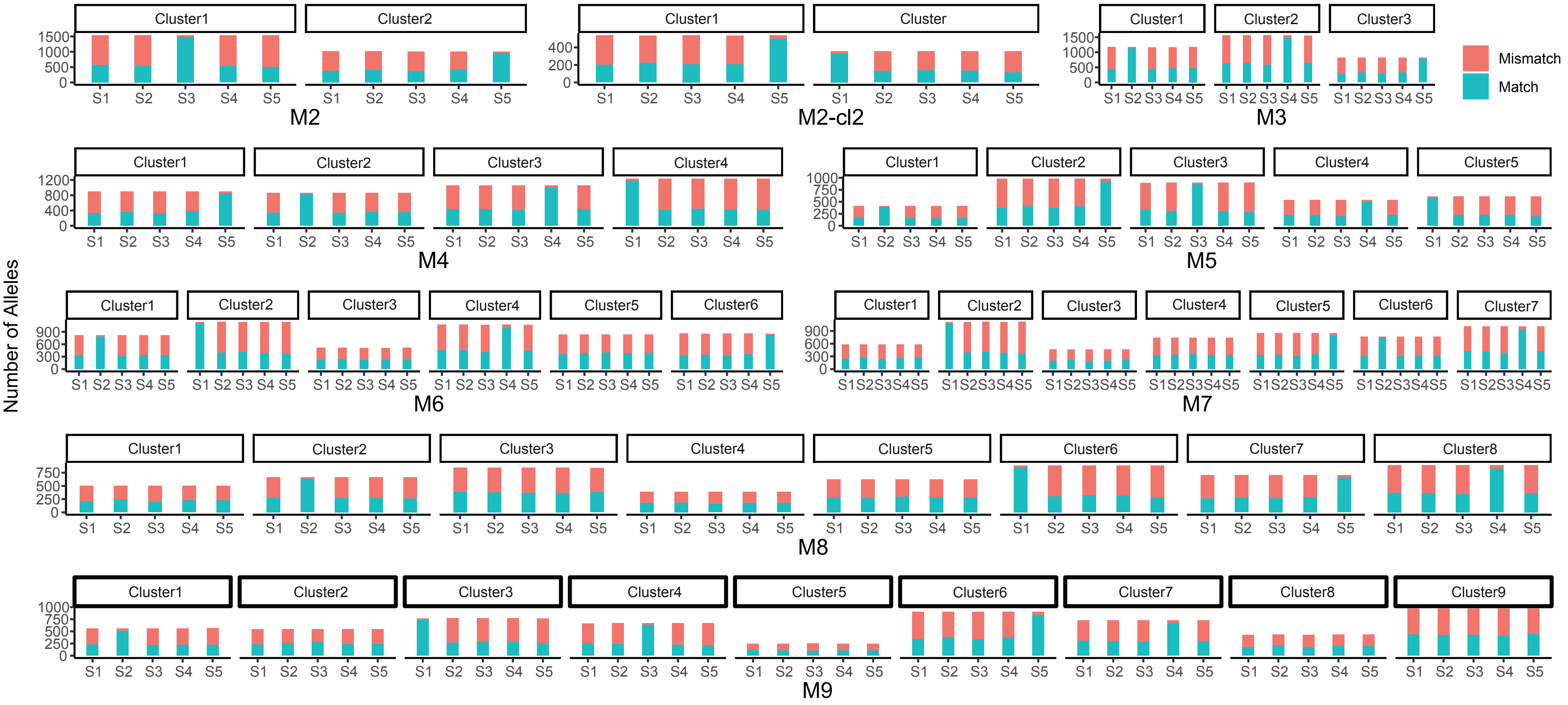

The comparison is made between the list of available SNPs per cluster in each deconvoluted mixture (M2-M9) and the whole exome sequencing reference. In each cluster that matches the whole exome sequencing (WES) reference we observe one sample matching with over 90% of the total SNPs while other samples maintain about 40% natural similarity towards to cluster. The number of available SNPs varies between the clusters depending on available SNPs in each cluster that also appears in the WES reference

Supplementary figure 7: The analysis of each cluster of the 4 person uneven mixture with 3 minor clusters.

a

Cluster	Y haplogroup	Quality	MT haplogorup	Quality
1	NA	NA	U5b2b4	0.8512
2	I2a1b1a2b1	1	T2a1a	0.7955
3	NA	NA	U5a2b4	0.7578
4	NA	NA	H11a1	0.9528

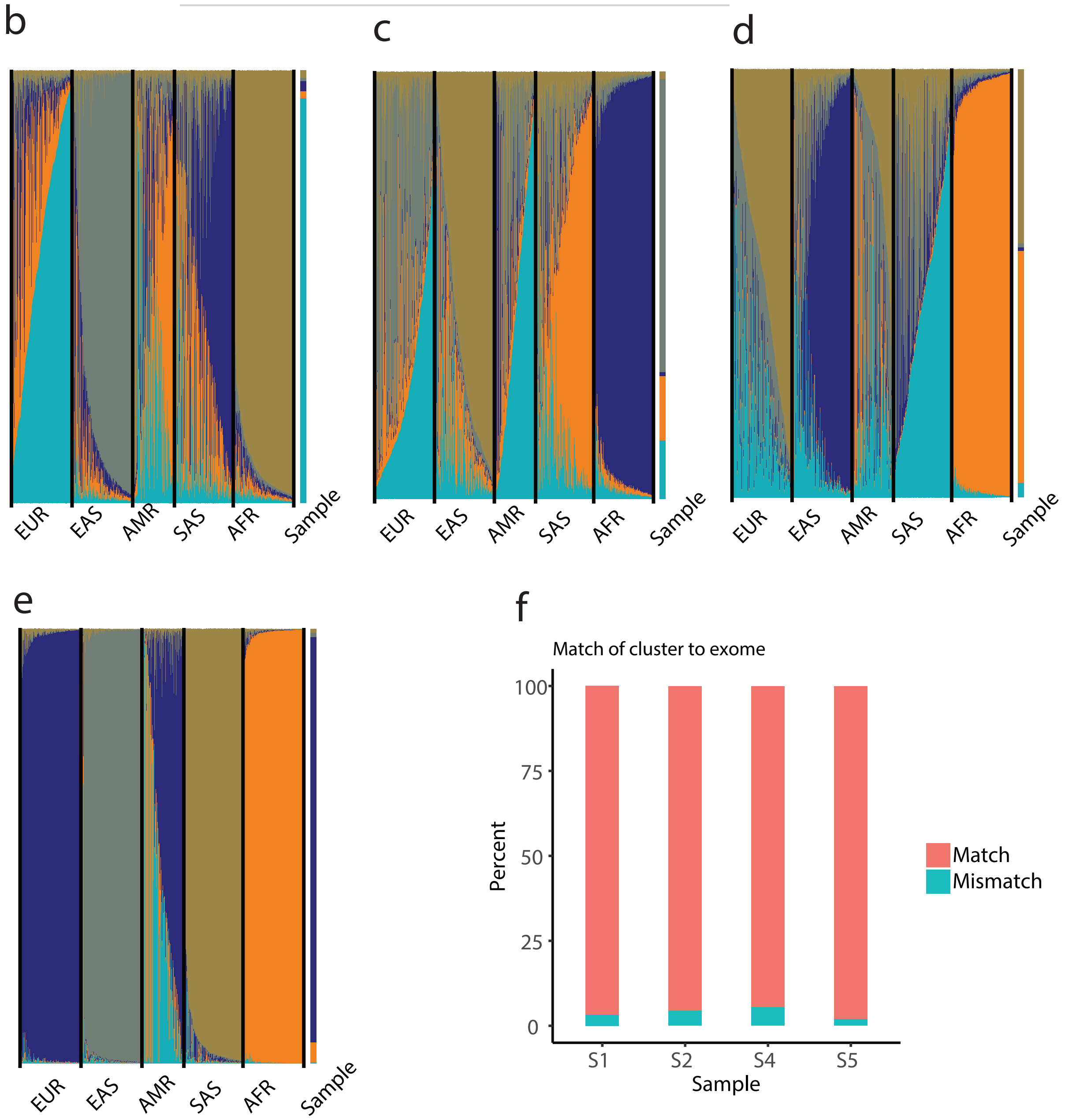

g

Cluster	LR	LR_B	PM	TotRM	Markers
1	454.09	2.54E+01	1.70E-03	2.20E-03	7
2	56.40	1.16E+01	1.11E-02	1.77E-02	5
3	66.51	8.03E+00	2.11E-02	1.50E-02	4
4	8.25E+42	1.29E+22	3.24e-41	1.21E-43	104

a) Results of Y chromosome (Yleaf) and mtDNA haplogroup assignment (Haplogrep). The three minor clusters (1-3) present relatively low confidence in the assigned haplogroup compared to the major cluster (4). One of the minor clusters is also missing a Y haplogroup. **b)** biparental ancestry of the minor cluster 1 in comparison to the ancestry prediction of samples with known ancestry (1000 Genomes). The prediction is correctly pointing towards European ancestry; however, the overall prediction of samples with known ancestry is unstable. We observe the same phenomena with minor cluster 2 (**c**) and minor cluster 3 (**d**). At the same time prediction for the major cluster 4 (**e**) is stable across the populations. **f)** Percentage match towards the correct exome sample for each cluster. Even with lower number of cells the matching ability stays stable. **g)** Calculation of LR and other forensic parameters. The limited number of markers in the minor clusters (1-3) provides only relatively low LR and PM compared to the major component cluster 4. The used depth per SNP is 20.

Supplementary figure 8: The analysis of each cluster of the 4 person uneven mixture with 1 minor cluster

a

Cluster	Y haplogroup	Quality	MT haplogorup	Quality
1	NA		H11a1	0.9528
2	I2a1b1a2b1a~	1	T2a1a	0.9324
3	NA		U5b2b4a	0.9824
4	E1b1a1a1	1	U5a2	0.824

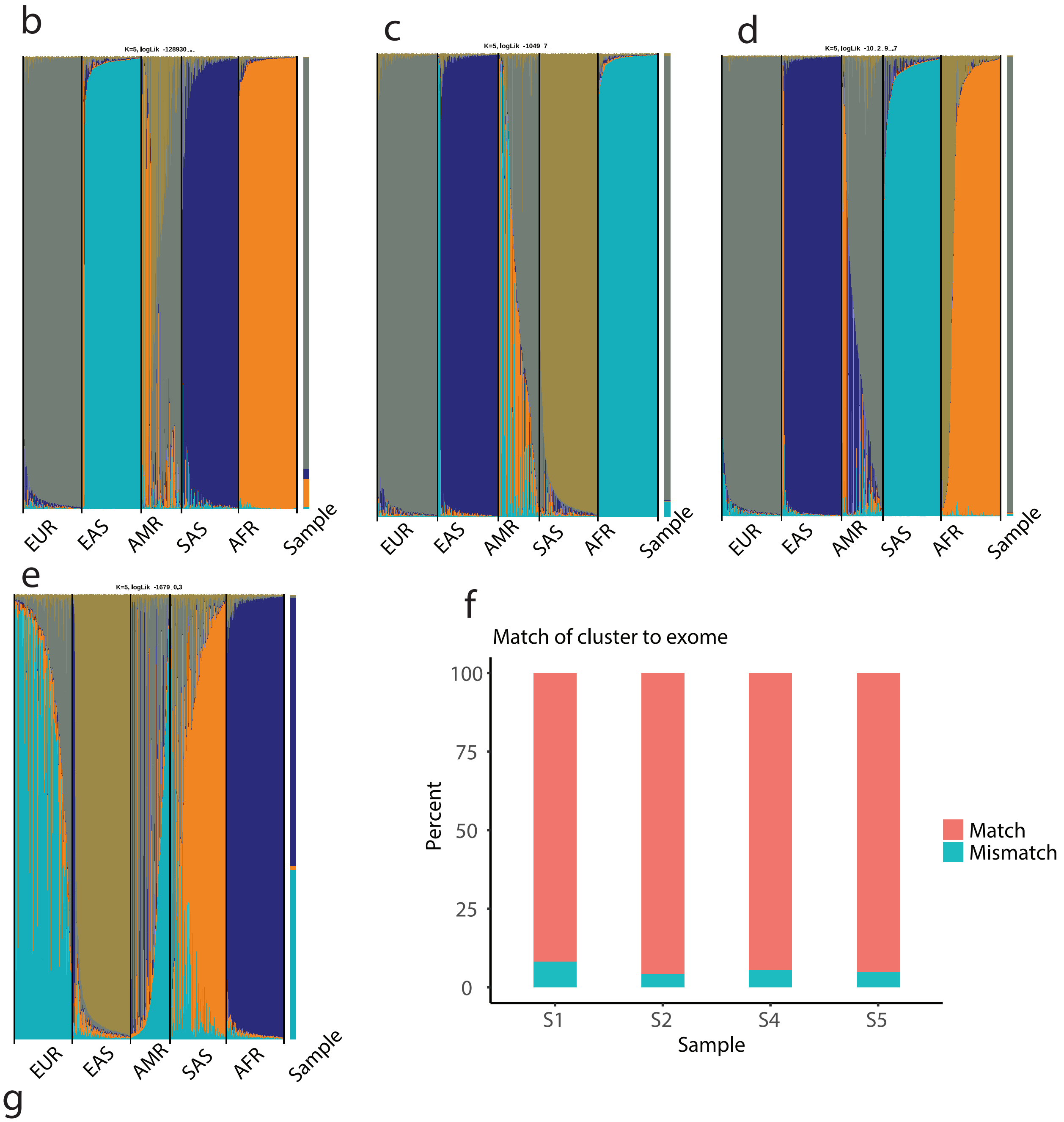

g

Cluster	LR	LR_B	PM	TotRM	Markers
1	5.04E+44	1.52E+23	3.79E-43	1.98E-45	109
2	4.18E+32	3.40E+17	8.87E-34	2.39E-33	85
3	6.14E+34	1.25E+18	6.81E-34	1.63E-35	86
4	2.05E+03	1.14E+02	7.54E-05	4.87E-04	10

a) Results of Y chromosome (Yleaf) and mtDNA haplogroup assignment (Haplogrep). The three major clusters (1-3) present high prediction confidence in the assigned haplogroup compared to the minor cluster (4). **b-d)** biparental ancestry of the major clusters 1-3 in comparison to the ancestry prediction of samples with known ancestry (1000 Genomes). The prediction is stable in between the populations as well as for the samples. **e)** biparental ancestry prediction of the minor cluster 4. The prediction of the sample correctly assigns mixed African and European ancestries. However, the overall stability of the prediction for the minor cluster is less reliable for the reference 1000 Genomes samples **f)** percentage match towards the correct exome sample for each cluster. Even with lower number of cells the matching ability stays stable. **g)** calculation of LR and other forensic parameters. The limited number of markers in the minor cluster (4) provides only relatively low LR and PM compared to the major component clusters 1-3. The used depth per SNP is 20.

Supplementary figure 9: Concordance of the separation of individuals for in silico mixture of 2 and in silico mixture of 9 individuals (previously used in the manuscript).

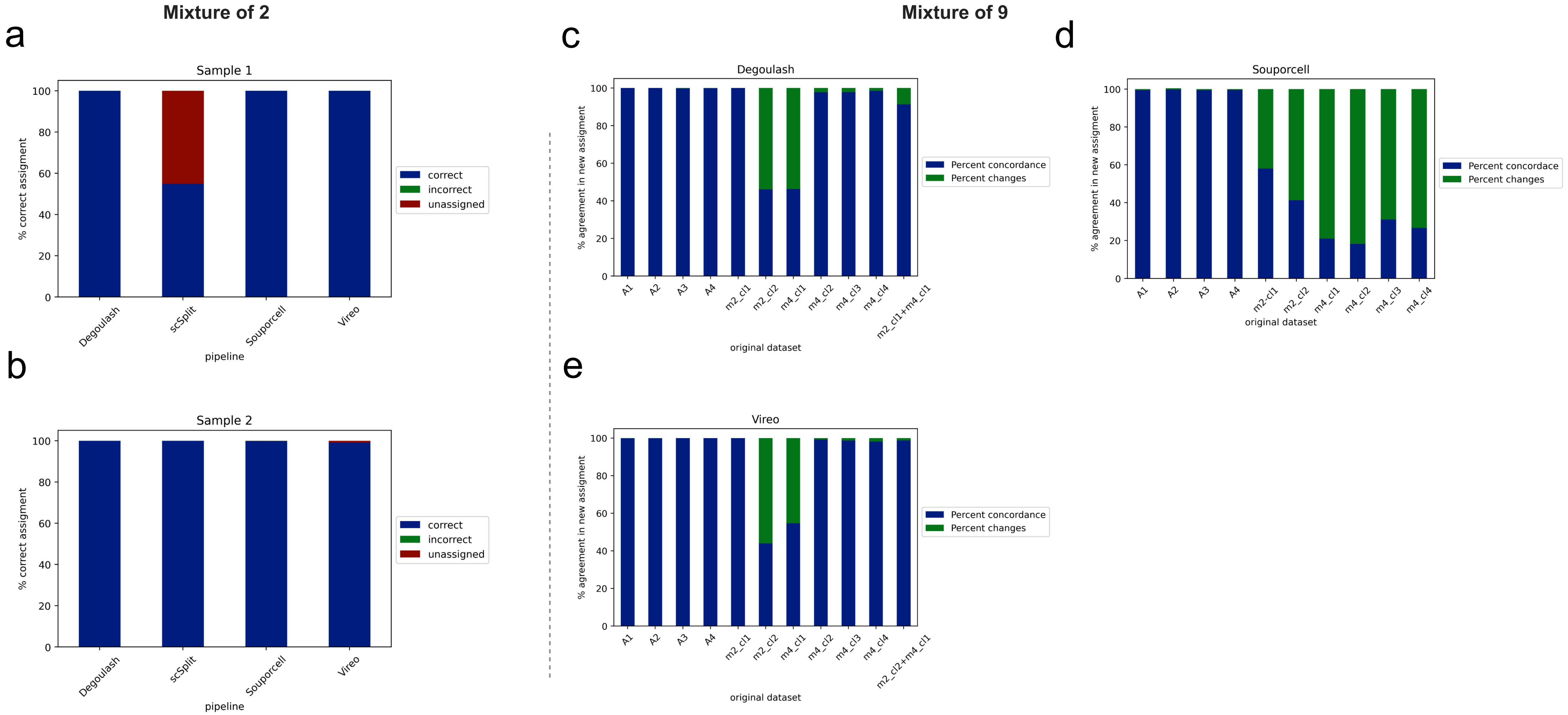

a) Results between the four pipelines (Degoulash, ScSplit, SoupOrCell, Vireo) for donor 1 (of 2) in mixture of 2 individuals. **b)** Results between the four pipelines (Degoulash, ScSplit, SoupOrCell, Vireo) for donor 2 (of 2) in mixture of 2 individuals. Since for mixture 2 we mixed 2 single source datasets we can determine the correct assignment of each cell reported in a and b. **c)** concordance results for mixture of 9 using the Degoulash pipeline. **d)** Concordance results for mixture of 9 using the SouOrCell pipeline. **e)** Concordance results for mixture of 9 using the Vireo pipeline. The mixture of 9 uses 4 single source datasets (A1-A4) and 2 biological mixture datasets (M2 and M4, mixtures of 2 and 4, respectively, with one shared individual between the clusters – M2-cluster2 and M4-cluster1). Therefore, we can determine the correct assignment for A1-A4 datasets. For clusters resulting from M2 and M4 we determine changes from the previous assignment (as reported in the manuscript and confirmed via the analysis pipeline and comparison with single source whole exome sequencing reference). ScSplit results are not reported here due to failure to finish citing insufficient memory on the large dataset.

Supplementary figure 10: The comparison of time and maximal RAM used during the run of various deconvolution pipelines.

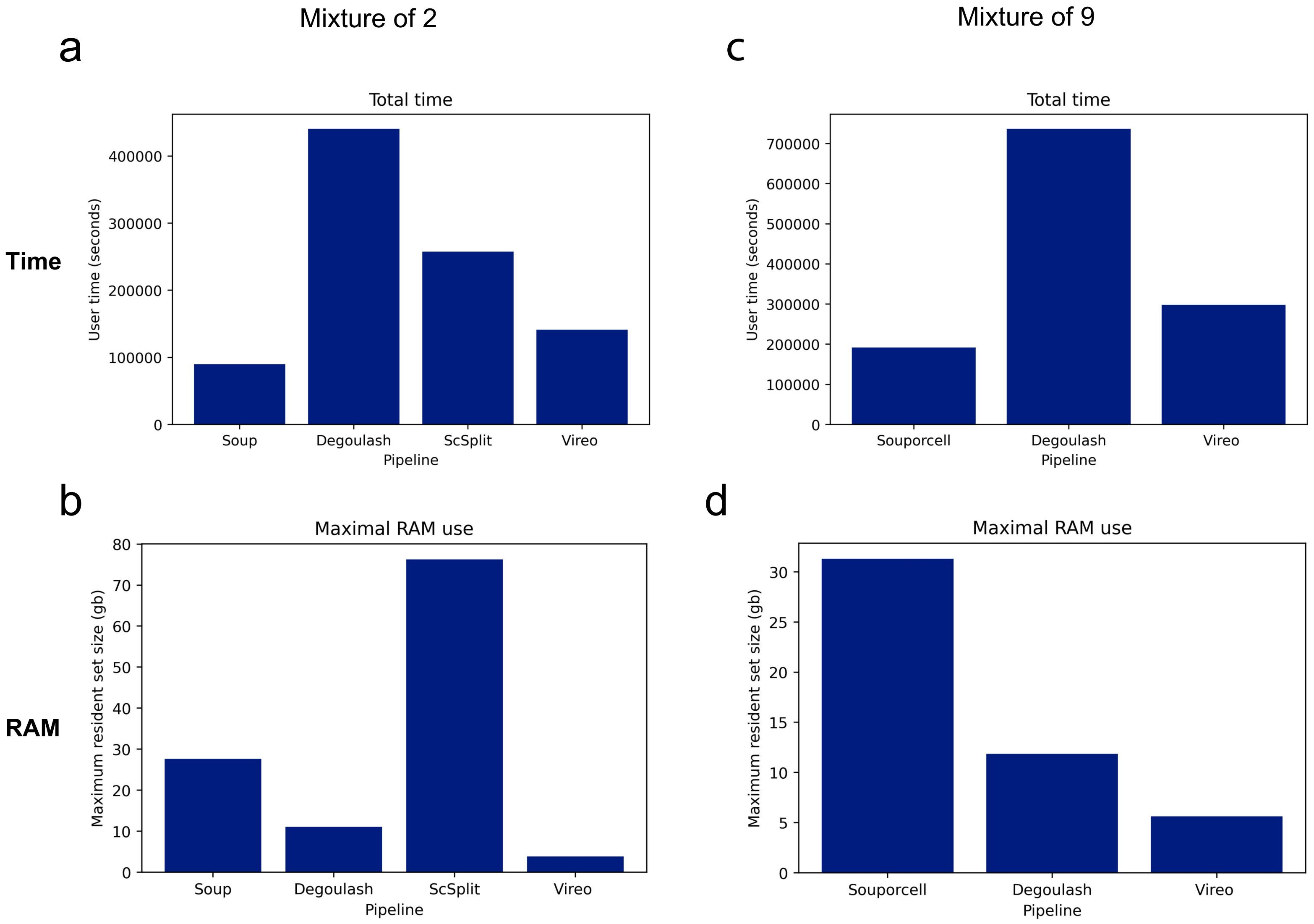

The mixtures used are a mixture of 2 individuals and a mixture of 9 individuals previously used in the manuscript. Due to some of the pipelines (Vireo, SoupOrCell) not supporting discovery mode (unknown number of contributing individuals) all pipelines were run with set number of individuals. This improved accuracy of Degoulash reported for the mixture of 9 in the manuscript. **a)** user time required for each pipeline with a in silico mixture of 2 individuals between SoupOrCell (Soup), Degoulash, ScSplit, and Vireo. **b)** The maximal resident set size in GB between the four pipelines for in silico mixture of 2 individuals. **c)** user time required for each pipeline for in silico mixture of 9 for SoupOrCell (Soup), Degoulash, and Vireo. ScSplit was not able to finish citing insufficient memory. **d)** The maximal resident set size in GB between the three pipelines for in silico mixture of 9 individuals. In a and b for scSplit the preprocessing of the dataset was added to the pipeline itself, for Vireo the preprocessing step using cellSNP is also added. This was done to provide the same starting point for each pipeline (bam file and barcode file).

Supplementary table 1. Separation of cells per mixture dataset in both iterations

Dataset	Iteration	SNPs	Cells	% of total cells
	Iter 1	62	2286	21.3
M2	Iter 2	630	10393	97.0
	Iter 1	13	647	7.5
M2-cl	Iter 2	256	6567	76.0
	Iter 1	59	4312	70.5
M3	Iter 2	475	5900	96.5
	Iter 1	67	5268	84.1
M4	Iter 2	1935	6118	97.6
	Iter 1	73	3508	43.9
M5	Iter 2	1107	7836	97.6
	Iter 1	49	1501	15.0
M6	Iter 2	1255	9245	92.5
	Iter 1	56	4648	46.5
M7	Iter 2	4486	9889	98.9
	Iter 1	42	724	7.2
M8	Iter 2	1253	9728	97.3
	Iter 1	51	3407	34.1
M9	Iter 2	1773	9817	98.2

Supplementary table 2. *In silico* mixture generation with regard to the dataset of origin and individual contribution in each mixture

	ID	M5	M6	M7	M8	M9
	N individuals	5	6	7	8	9
Dataset used	M2	X				X
	M4	X	X	X	X	X
	A1		X	X	X	X
	A2				X	X
	A3			X	X	X
	A4		X	X	X	X
	Cells per individual	1600	1667	1429	1250	1000

Notes: M2 and M4 are biological mixtures of 2 and 4 individuals respectively. The mixtures share one individual between them (S5). A1-A4 are publically available single source datasets. M5 was made with 5000 cells from M2 and 3000 cells from M4 to correct on difference between the mixtures regarding ratios and reads per cell.

Supplementary table 3. Y chromosome and mtDNA haplogroup assignment reference WES sequencing (S1-S5) and publicly available single-origin sc-RNA datasets

Sample	Y haplogroup	QC score	MT haplo	QC score
S1	E1b1a1a1	1	NA	NA
S2	NA	0	NA	NA
S3	E1b1b1b2a1a1	1	NA	NA
S4	NA	NA	NA	NA
S5	NA	NA	NA	NA
A2	R1b1a1b1a1a2b	0.956	V1a1a1	0.95
A3	R1b1a1b	0.978	K1a4a1	0.96
A4	Q2a1a	0.964	X2c1a	1
A1	NA	Na	E1a1a1	0.97

Supplementary table 4. Biparental ancestry determination towards the major population determined by 1000 Genomes for the reference WES sequencing (S1-S5) and publicly available single-origin scRNA-seq datasets (A1-A4)

Sample	Eur	Sas	Amr	Eas	Afr
S1	0.299	0.015	0.003	0.01	0.673
S2	0.914	0.039	0.014	0.01	0.023
S3	0.342	0.021	0.029	0.056	0.557
S4	0.961	0.006	0.009	0.012	0.012
S5	0.953	0.023	0.016	0.005	0.002
A2	0.485	0.115	0.132	0.112	0.156
A3	0.685	0.061	0.023	0.121	0.111
A4	0.571	0.069	0.082	0.142	0.135
A1	0.092	0.71	0.023	0.085	0.09

Supplementary table 5. Reference whole exome sequencing description in terms of quality, depth, and composition of each sample

Sample	Raw reads	Aligned reads	Duplicate percentage	Usable reads	On-target bases	Mean coverage	1x	5x	10x	15x	20x	25x	30x	35x	40x	50x	100x
S1	33292862	33197548	0.163412	27772670	2751122930	59.06	99.7	99.1	97.3	93.9	88.7	82.1	74.8	67.4	60.1	47	13.4
S2	50421126	50279374	0.170668	41698293	4082511474	87.64	99.8	98.8	96	91.5	86.1	80.4	74.8	69.5	64.7	56.2	30.5
S3	35615738	35517096	0.167061	29583574	2927585500	62.85	99.7	98.8	95.9	91.2	85.2	78.7	72	65.6	59.5	48.6	17.1
S4	28621266	28462198	0.167976	23681231	2101159799	45.1	99.5	98.4	94.8	88.5	80.2	71.1	62	53.5	45.8	32.8	6.2
S5	60872222	60690152	0.184995	49462777	4822816371	103.53	99.6	99.3	98.6	97.2	94.8	91.6	87.7	83.4	78.8	69.8	37

Supplementary table 5 continued. Reference whole exome sequencing description in terms of quality, depth, and composition of each sample

Sample	FreeMix	RawVariants	RawNovels	RawcompRatedbSNP	RawTiTv	RawHetHom	Yield	Unaligned	Duplicates	OffTarget	Output
S1	0.00536	50711	6579	87.03	2.45	2.85	4.9939293	0.0142971	0.8137316571	1.414777613	2.75112293
S2	0.00578	45284	6776	85.04	2.32	2.15	7.5631689	0.0212628	1.28716203	2.172232596	4.082511474
S3	0.0041	49037	6962	85.8	2.47	2.35	5.3423607	0.0147963	0.8900282362	1.509950664	2.9275855
S4	0.00672	50882	12396	75.64	2.02	2.58	4.2931899	0.0238602	0.7171449257	1.451024975	2.101159799
S5	0.00442	42399	3513	91.71	2.57	1.95	9.1308333	0.0273105	1.6841062	2.596600229	4.822816371

Supplementary table 6. Uneven mixture cell composition

Mixture	A2	A4
1:9	100	900
1:20	50	950
1:40	25	975
1:60	17	983
1:80	13	987
1:90	10	990

Supplementary table 7. Comparison of the uneven mixture deconvolution to the datasets of origin

Dataset	Cluster	Correct	Incorrect	Separated cells
	1	100	0	
1:10	2	881	0	981
	1	50	0	
1:20	2	927	0	971
	1	956	0	
1:40	2	25	0	981
	1	16	0	
1:60	2	903	0	956
	1	0	15	
1:80	2	941	13	980
	1	65	0	
	2	73	1	
	3	76	1	
1 major	4	1971	42	2187
	1	1978	38	
	2	1217	6	
	3	1307	5	
3 major	4	152	3	4656

Supplementary table 8. Uniparental ancestry, biparental ancestry and forensic parameters for the minor components of uneven mixtures of higher degree

Cluster	Cells	Y haplogroup	Y Qual	MT	MT QUAL	Structure	match	LR	LR_B	PM	TotRM	Power of exclusion	Power of discrimination	markers
1 minor	152	E1b1a1a1	0.96	R	0.8676	Afr+Eur	0.885	6.87E+08	8.02E+04	3.02E-10	1.46E-09	1	1	24
3 minor -1	66	NA	NA	U5b2b4	0.9622	Eur+Sas	0.958	2.14E+04	2.2E+02	1.57E-05	4.67E-05	0.993238	0.999984	12
3 minor -2	76	I2a1b1a2b1	1	T2a1a	0.8212	Eur	0.933	1.26E+03	6.39E+01	2.56E-04	7.96E-04	0.976419	0.999744	9
3 minor -3	73	E1b1a1a1	1	U5a2b4	0.8309	Eur+Afr	0.948	2.35E+03	4.52E+01	1.38E-03	4.25E-04	0.950701	0.998623	7

Supplementary table 9. Markers matching towards the exome reference per cluster and number of cells

Cluster	Cells	Average Match	St.dev. Avr match	Average Markers	St. dev avr.markers
	10	0.8359	0.1524	12.40	3.8644
	20	0.9500	0.0284	28.13	3.7961
	30	0.9564	0.0162	45.90	6.3675
	50	0.9638	0.0168	71.40	5.1251
	100	0.9710	0.0135	139.90	11.6471
	150	0.9665	0.0091	214.70	9.1415
	200	0.9634	0.0101	287.80	12.2728
	300	0.9692	0.0105	447.56	20.1997
	400	0.9633	0.0071	580.30	21.4841
Cluster 1	500	0.9649	0.0066	720.30	21.6490
	10	0.9864	0.0290	11.60	4.0607
	20	0.9658	0.0473	22.20	3.4577
	30	0.9750	0.0265	33.30	5.3344
	50	0.9497	0.0507	58.30	9.6154
	100	0.9529	0.0366	116.50	10.9671
	150	0.9597	0.0134	176.50	4.6248
	200	0.9353	0.0122	272.30	9.2502
	300	0.9489	0.0121	376.90	16.4212
	400	0.9433	0.0119	510.20	21.1755
Cluster 2	500	0.9466	0.0098	635.20	11.1036
Cluster 3	10	0.9676	0.0438	11.50	4.5826

	20	0.9481	0.0650	26.40	3.7859
	30	0.9655	0.0320	42.30	4.1633
	50	0.9615	0.0364	66.40	4.3589
	100	0.9566	0.0145	124.30	14.3643
	150	0.9498	0.0160	203.70	8.5049
	200	0.9353	0.0122	272.30	11.1355
	300	0.9262	0.0106	511.90	71.1899
	400	0.9354	0.0131	529.40	26.3122
	500	0.9325	0.0071	644.80	21.5948
	10	0.9250	0.0570	11.57	2.2991
	20	0.9084	0.0747	24.86	5.3675
	30	0.9037	0.0489	37.00	4.4721
	50	0.9180	0.0370	55.14	3.9340
	100	0.9219	0.0245	102.14	1.6762
	150	0.9230	0.0179	149.14	9.9067
	200	0.9210	0.0122	205.71	9.4112
	300	0.9214	0.0132	307.00	11.2398
	400	0.9241	0.0067	432.29	14.6937
Cluster 4	500	0.9221	0.0077	533.43	9.0895
