# Supplementary material for: Single-cell transcriptome sequencing allows genetic separation, characterization and identification of individuals in multi-person biological mixtures
Source: Commun Biol. 2023 Feb 20;6:201. doi: 10.1038/s42003-023-04557-z (PMC9941516; doi:10.1038/s42003-023-04557-z)
Supplement: Supplementary file 2 — Description of Additional Supplementary Files [file 42003_2023_4557_MOESM2_ESM.pdf]

Description of Additional Supplementary Files

File name: Supplementary Data 1
Description: Summary of sequencing quality and depth per dataset.

File name: Supplementary Data 2
Description: Percentage of reads aligned to Y chromosome per assigned cluster in each dataset.

File name: Supplementary Data 3
Description: Percentage of reads on X chromosome and in the XIST gene compared to the Yleaf results per assigned cluster in each dataset.

File name: Supplementary Data 4
Description: Paternal and Maternal ancestry prediction after mixture deconvolutio comparison to the available reference data.

File name: Supplementary Data 5
Description: Biparental ancestry assessment in each cluster of the deconvoluted mixtures towards five major populations assigned by 1000 Genomes.

File name: Supplementary Data 6
Description: Allele match toward the exome reference per cluster after mixture deconvolution for each deconvoluted mixture.

File name: Supplementary Data 7
Description: Calculation of forensic parameters per cluster after mixture deconvolution.

File name: Supplementary Data 8
Description: Calculation of forensic parameters per cluster and number of cells.

File name: Supplementary Data 9
Description: Determination of Y chromosome haplogroup per number of cell per cluster.

File name: Supplementary Data 10
Description: Determination of mtDNA haplogroup per cluster and number of cells.
