# Supplementary material for: Single-cell transcriptome sequencing allows genetic separation, characterization and identification of individuals in multi-person biological mixtures
Source: Commun Biol. 2023 Feb 20;6:201. doi: 10.1038/s42003-023-04557-z (PMC9941516; doi:10.1038/s42003-023-04557-z)
Supplement: Supplementary file 13 — Reporting summary [file 42003_2023_4557_MOESM13_ESM.pdf]

nature portfolio

Corresponding author(s): Manfred Kayser
Eskeatnaf Mulugeta

Last updated by author(s): 27/01/2023

Reporting Summary

Nature Portfolio wishes to improve the reproducibility of the work that we publish. This form provides structure for consistency and transparency in reporting. For further information on Nature Portfolio policies, see our Editorial Policies and the Editorial Policy Checklist.

Statistics

For all statistical analyses, confirm that the following items are present in the figure legend, table legend, main text, or Methods section.

n/a	Confirmed	
☐	☒	The exact sample size (*n*) for each experimental group/condition, given as a discrete number and unit of measurement
☐	☒	A statement on whether measurements were taken from distinct samples or whether the same sample was measured repeatedly
☐	☒	The statistical test(s) used AND whether they are one- or two-sided<br>*Only common tests should be described solely by name; describe more complex techniques in the Methods section.*
☐	☒	A description of all covariates tested
☒	☐	A description of any assumptions or corrections, such as tests of normality and adjustment for multiple comparisons
☐	☒	A full description of the statistical parameters including central tendency (e.g. means) or other basic estimates (e.g. regression coefficient) AND variation (e.g. standard deviation) or associated estimates of uncertainty (e.g. confidence intervals)
☐	☒	For null hypothesis testing, the test statistic (e.g. *F*, *t*, *r*) with confidence intervals, effect sizes, degrees of freedom and *P* value noted<br>*Give P values as exact values whenever suitable.*
☒	☐	For Bayesian analysis, information on the choice of priors and Markov chain Monte Carlo settings
☐	☒	For hierarchical and complex designs, identification of the appropriate level for tests and full reporting of outcomes
☒	☐	Estimates of effect sizes (e.g. Cohen's *d*, Pearson's *r*), indicating how they were calculated

Our web collection on statistics for biologists contains articles on many of the points above.

Software and code

Policy information about availability of computer code

Data collection

No software used for data collection. Publicly available scRNA-seq datasets were obtained from 10x genomics (https://www.10xgenomics.com/resources/datasets).

Data analysis

We have developed a Bioinformatic pipeline for this specific study. The bioinformatics pipeline de-goulash will be made available at: https://github.com/genid/de-goulash (available after acceptance of the manuscript, reviewers can get access)

Single cell RNA-seq data
Sequencing reads were aligned to the human genome (GRCh38) with the STAR aligner that is part of the Cell Ranger 3.0.2 software (10X Genomics).
Aligned scRNA-seq data (BAM file) was filtered using two criteria with subset-bam v1.1.0
BAM file was indexed and sorted by TAG using samtools v.1.9[41] and split into individual cell BAM files with a custom made Pysam v0.15.4.
Variants were called (on the whole dataset BAM file) with parallel FreeBayes v1.3.1
Cluster variant lists were merged using Picard Tools version 2.25.6 MergeVcfs.

Whole exome sequencing data
Whole exome sequencing data aligned to the human genome reference hg19 using the Burrow-Wheeler alignment tool (BWA version 0.7.3a).Base quality score was recalibrated and indels realigned using Genome Analysis ToolKit (GATK version 3.7). Duplicates were marked using Picard (Picard Tools version 1.90). Variant calling was performed with HaplotypeCaller (GATK v3.8). Subsequently, the samples were pooled for combined calling with GATKs GenotypeVCFs and VariantQualityScoreRecalibration workflow.

Genetic characterisation analyses

Maternal (mtDNA) ancestry was acquired by applying Haplogrep2.1.20 .
The Y chromosome ancestry was determined using Y-leaf.
Autosomal ancestry was determined using STRUCTURE.

For manuscripts utilizing custom algorithms or software that are central to the research but not yet described in published literature, software must be made available to editors and reviewers. We strongly encourage code deposition in a community repository (e.g. GitHub). See the Nature Portfolio guidelines for submitting code & software for further information.

Data

Policy information about availability of data

All manuscripts must include a data availability statement. This statement should provide the following information, where applicable:

- Accession codes, unique identifiers, or web links for publicly available datasets
- A description of any restrictions on data availability
- For clinical datasets or third party data, please ensure that the statement adheres to our policy

The individual datasets used in the in-silico part of the study are available via the 10x website:
A1: https://www.10xgenomics.com/resources/datasets/5-k-peripheral-blood-mononuclear-cells-pbm-cs-from-a-healthy-donor-v-3-chemistry-3.0.2
A2: https://www.10xgenomics.com/resources/datasets/peripheral-blood-mononuclear-cells-pbm-cs-from-a-healthy-donor-chromium-connect-channel-1-3.1.0
A3: https://www.10xgenomics.com/resources/datasets/4-k-pbm-cs-from-a-healthy-donor-2.1.0
A4: https://www.10xgenomics.com/resources/datasets/10-k-pbm-cs-from-a-healthy-donor-gene-expression-and-cell-surface-protein-3.0.0
The mixture datasets that were de-novo generated in this study are available at the EGA via restricted access (data available after approval by Data Access Committee)
database with EGAS00001006202.

Human research participants

Policy information about studies involving human research participants and Sex and Gender in Research.

Reporting on sex and gender	We collected information about biological sex, paternal and maternal ancestry, and forensically relevant appearance traits (hair color, skin color, eyebrow color, eye color) of each individual involved in the study. The findings apply or the technique we developed can be applied for determining sex (genetic). All data was collected via a self-reporting questionnaire.
Population characteristics	All individuals involved were healthy volunteers above the age of 18. For the method we are developing, consent and being above 18 were the only necessary criteria.
Recruitment	The volunteers were recruited via internal call for study volunteers within the institution. The selection of individuals was done based on their biological sex and presumed (self-reported) ancestry to ensure variable backgrounds in the study.
Ethics oversight	The study was carried out in compliance with research rules and regulations at Erasmus MC including those on ethics by the Medical Ethics Committee (METC) of Erasmus MC. A consent form was provided by all volunteers involved.

Note that full information on the approval of the study protocol must also be provided in the manuscript.

Field-specific reporting

Please select the one below that is the best fit for your research. If you are not sure, read the appropriate sections before making your selection.

☒ Life sciences ☐ Behavioural & social sciences ☐ Ecological, evolutionary & environmental sciences

For a reference copy of the document with all sections, see nature.com/documents/nr-reporting-summary-flat.pdf

Life sciences study design

All studies must disclose on these points even when the disclosure is negative.

Sample size	Sample size was determined considering a reasonable forensic scenario and in-silico testing of the analysis. We determined the number of participants in the study accompanied by publicly available data to be sufficient to attest the functionality and strength of the approach.
Data exclusions	No dataset was excluded from the analysis. Sequenced cells that presented a low amount of SNPs to be successfully clustered to an individual were excluded from further downstream analysis.
Replication	The reproducibility of the approach was tested using different approaches. First, using two individuals and then by increasing the number of

Replication	individuals to 4. These samples were then deconvoluted using the same approach. Furthermore, we used independent, publicly available data to test the reproducibility as well and functionality of our approach.
Randomization	Each dataset comprises of multiple individuals. The individuals were selected based on their reported population background to ensure validation of the presented approach on variable samples with different number of individuals and varying (similar and distant) background.
Blinding	Due to the intended identification of individuals from each dataset complete blinding of the study was not possible. For validation purposes the investigator was provided with a reference for each individual present in the dataset as well as the presumed ancestry. This information was then used to validate the correct deconvolution of each mixture and asses the downstream analysis.

Reporting for specific materials, systems and methods

We require information from authors about some types of materials, experimental systems and methods used in many studies. Here, indicate whether each material, system or method listed is relevant to your study. If you are not sure if a list item applies to your research, read the appropriate section before selecting a response.

Materials & experimental systems

n/a	Involved in the study
☒	☐ Antibodies
☒	☐ Eukaryotic cell lines
☒	☐ Palaeontology and archaeology
☒	☐ Animals and other organisms
☒	☐ Clinical data
☒	☐ Dual use research of concern

Methods

n/a	Involved in the study
☒	☐ ChIP-seq
☒	☐ Flow cytometry
☒	☐ MRI-based neuroimaging

March 2021
